# Significant strain microdiversity in mother-infant dyad cohorts across ethnic groups reveals population specificity of bifidobacteria microbiota transmission

**DOI:** 10.3389/fmicb.2026.1814222

**Published:** 2026-05-29

**Authors:** Huimin Zhang, Quanhao Zhao, Baolong Luo, Xueling Zhang, Jian Huang, Yanzhuan Lu, Fengwei Tian, Hailong Sun, Yongqing Ni

**Affiliations:** 1 School of Food Science and Technology/Key Laboratory for Xinjiang Special Probiotics and Dairy Technology of the Eighth Division of XPCC, Shihezi University, Shihezi, China; 2 School of Food Science and Technology, Jiangnan University, Wuxi, China

**Keywords:** *Bifidobacterium*, gut microbiota, mother-infant dyads, population specificity, transmission

## Abstract

The gut microbiota of human populations shares a core of symbiotic microbial species, some of which codiversify with hosts, and are considered a complex mixture of closely related strains. However, little is known about population-wide diversity for strain-level symbiont community in the human gut so far. Here, we focused on *Bifidobacterium*, a key microbial group in the early-life gut microbiota. By analyzing metataxonomic datasets of the full-length 16S rRNA gene and the *Bifidobacterium*-specific *groEL* and *tuf* genes from 54 mother-infant dyads across three ethnic groups spanning large geographic distances in China, we determined that 16S rRNA gene primer sequencing causes significant deviations in species and strain diversity of the Bifidobacteria community. In the single-copy *groEL* nd *tuf* gene dataset, a core group comprising at least 10 bifidobacterial (sub)species was consistently identified across multiple cohorts. ASVs within the same species represent significant microdiversity, showing distinct distribution patterns across cohorts. Notably, strain similarity within a cohort was significantly higher than that across cohorts, supporting the hypothesis of population specificity in intergenerational inheritance of gut symbiotic consortia within sympatric populations.

## Introduction

The gut microbiota and human host mutually affect and depend on each other in an intimate relationship, so humans are considered a holobiont from a holistic view of this association (Kurilshikov et al., 2021; Chen et al., 2025; Zeng et al., 2026). Although human gut microbiota seems to be made up of thousands of microbial species, a considerable part of the members were introduced into the human body through environmental exposure and diet, not persistent gut residents; only a fraction of the gut microbes have established a mutually symbiotic relationship with the host (Zilber-Rosenberg and Rosenberg, 2021). It was these microbes that are among the key factors in regulating host health, boosting immune system maturation, and directly protecting the host from pathogen infection (Groussin et al., 2020; Rook and Zwart, 2025; Qi et al., 2022).

Bifidobacteria are a paragon of symbiotic bacteria in the human gut that have accompanied human beings since the ape-man stage and have evolved a high degree of host specialization (Groussin et al., 2020; Lugli et al., 2020; Nishida and Ochman, 2019; Reese et al., 2021). The species that mostly occur in the human host are generally referred to as Human-Residential Bifidobacteria (HRB; Wong et al., 2020). Nowadays, we know that some HRB are highly abundant colonizers in the early stages of human life, and their presence is associated with many beneficial host effects that can persist throughout life (Laursen and Roager, 2023; Afzaal et al., 2022; Xiao et al., 2024). Disruption of the microbiota and abnormality of HRB early in life may increase the risk of disease in childhood or later in life (Afzaal et al., 2022; Mujagic et al., 2022; Underwood et al., 2020). The consensus so far is that *Bifidobacterium* lineages in the infant gut are vertically transferred from the maternal gut through breastfeeding (Browne et al., 2022). After being seeded with breast milk, the occurrence and abundance of different HRB species in the human gut depend on the type of carbohydrate consumed in the human diet (Laursen and Roager, 2023; Zhao et al., 2025). Among the HRB documented, *B. bifidum*, *B. breve*, and *B. longum* subsp. *Infantis*, termed as infant-type, have a superior ability to utilize HMOs (human milk oligosaccharides), leading to their high abundance and prevalence in infant intestines (Luo et al., 2021; Liu et al., 2026; Lin et al., 2025). Conversely, *B. adolescentis*, *B. catenulatum*, and *B. pseudocatenulatum*, *which are* representative of adult-type HRB, tend to have a greater ability to metabolize dietary sources of glycans, giving them an advantage in the adult gut (Maher et al., 2020).

Gut symbionts rely on their host to maintain a stable habitat environment and access nutrients (Xiao et al., 2024; Culp and Goodman, 2023). Within a species, different genotypes exhibit remarkable population-specific distribution patterns across human populations (Van Rossum et al., 2020). Different genotypes within the same *Bifidobacterium* species may exhibit distinct degradation capabilities toward human milk oligosaccharides (HMOs) and/or dietary plant-derived glycans, which can vary according to their original human population sources (Singh, 2019; Zhang et al., 2022). Based on current understanding of mother-to-infant microbial transmission, the *Bifidobacterium* composition in infants should correspond to and be consistent with that in their mothers, despite considerable differences in relative abundance. Therefore, the conspecific strains of both infant-type or adult-type bifidobacteria species from the same mother-infant pair would experience the same selective pressure and share similar genetic architecture (same or similar strain combination; Yan et al., 2021; Flores Ventura et al., 2025). Furthermore, in numerous instances, HMOs and dietary glycans are only partially degraded by certain symbiont bacterial strains, leading to a prevalent cross-feeding interaction within both maternal and infant gut microbiota, particularly between bifidobacterial species/strains (Luo et al., 2021; Culp and Goodman, 2023). Cross-feeding interactions contribute to genotypic diversity (at the strain level) within natural microbial populations under particular nutrient availability (Culp and Goodman, 2023). Indigenous people within the same geographical region often share similar dietary habits, leading to an enrichment of similar symbiont microbial species/strain profiles (Lu et al., 2021; Cheng et al., 2021). Consequently, we have a basis to hypothesize that certain symbiont lineages displaying unique genetic traits may be confined to specific dietary ethnic groups inhabiting a limited geographical area, thereby resulting in the emergence of distinct strain profiles characterized by population-specific characteristics associated with ethnicity, shared living environment, and cultural practices. Understanding the symbiont composition of human population-specific signatures will aid in developing therapeutics for targeted modulation of microbiota, especially at the strain level (Dehingia et al., 2019; Sun et al., 2024).

However, *Bifidobacterium* is not the dominant bacterium in the adult gut compared to the infant gut microbiota, with a relative abundance of only 1% or less, and even undetected in the gut of some populations (Derrien et al., 2022). Particularly, the pitfalls inherent to 16S subregions, such as V4 region or V3-V4 region which are the most frequently used markers in the studies profiling the human gut microbiota, could lead to the under-representation of the relative abundance of bifidobacterial taxa in the sequence dataset and the difficulty in identifying bifidobacteria community to the species level (Callahan et al., 2019; Walker et al., 2015; Alcon-Giner et al., 2017). As a result, although humans share many of the same *Bifidobacterium* species in their gut microbiota across populations, 16S rRNA gene amplicon studies reported the conspicuous inter-cohort inconsistency for the abundance and prevalence of HRB species in the infant’s gut and corresponding mother’s gut (Alessandri et al., 2021; Casaburi et al., 2021). In particular, current conclusions about the occurrence and association of bifidobacterial populations between mothers and infants remain ambiguous at the strain level.

In this study, we utilized cultured strains and metataxonomic datasets of the full-length 16S rRNA gene for all bacteria, in conjunction with *groEL* and *tuf* gene specific to *Bifidobacterium*, to characterize the gut microbiota of 54 mother-infant dyads (two stages of lactation, namely newborn and 6-month-old infants) from three distinct ethnic groups in China, the habitats of which are separated by more than 2,000–4,000 km, with contrasting geographical and climatic environments. To achieve data reliability, using three mock communities (MC) from mixtures of different proportions of strains belonging to five *Bifidobacterium* species (subspecies), the *groEL* and *tuf* genes sequencing were evaluated to determine whether they can truly represent the composition of the Bifidobacteria community. We aimed to gain insight into the diversity and composition of bifidobacteria community in mother-infant dyads across different ethnic groups, and to determine whether there is ethnic specificity in *Bifidobacterium* community transmitted by analyzing temporal dynamic of HRB taxa at the species- and strain-level across three mother-infant dyads cohorts.

## Materials and methods

### Subject selection and fecal sample collection

**Figure 1:**
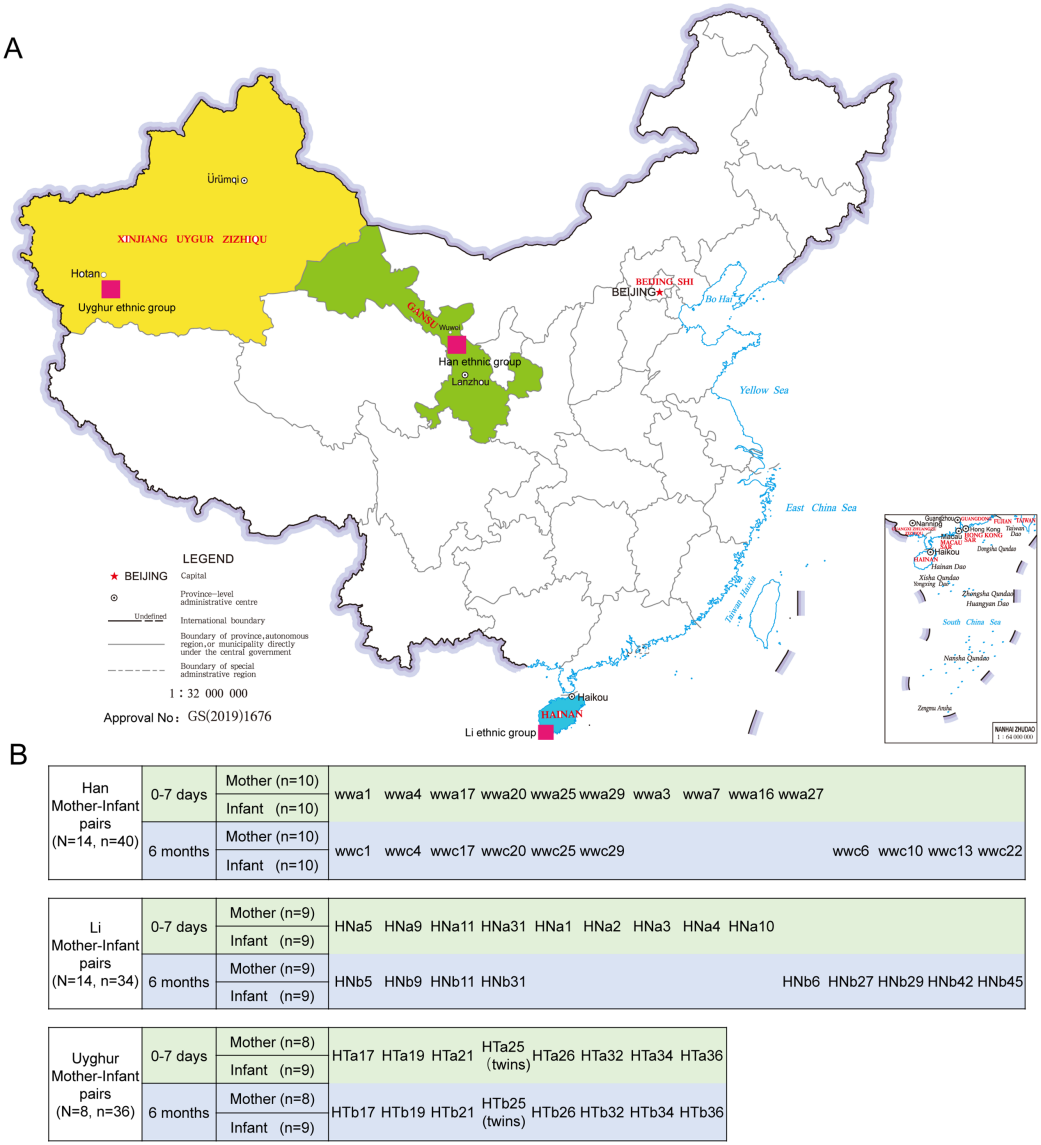
Sample distribution statistics. **(A)** Schematic map of sampling locations. **(B)** Sample information and longitudinal cohort of mother-infant pairs from three ethnic populations. *N* represents the number of individuals, and *n* represents the number of samples.

In this study, a total of 54 mother-infant pairs were selected, among which one pair was twins (two periods of lactation, including 7 days after birth and 6-month-old infants) from the Han in Gansu, Li in Hainan, and Uyghur in Xinjiang, China (Figures 1A,B). All volunteers recruited in this study were local inhabitants and retained the custom of strict intra-ethnic marriages. Specifically, the Uyghur ethnic group has long resided in Xinjiang in northwestern China. They mostly reside in compact communities and are primarily engaged in agricultural activities. Their daily diet was dominated by wheat-based foods (naan bread, noodles), pilaf, nuts, and fermented dairy products, with relatively low intake of vegetables and fruits. The Han ethnic group has long been resident in Gansu Province, China, approximately 2,000 km from Xinjiang. This region is characterized by a dry climate with little rainfall year-round. Their diet was relatively diverse, with staple foods including rice and wheat-based products (such as beef noodles and steamed buns), as well as milk and fruits. The Li ethnic group resided in Hainan Island, China, about 2,000 km from the Han community in Gansu and roughly 4,000 km from Xinjiang. This region is coastal, with a warm, humid climate and abundant seafood and fruits. Residents had lived on the island for generations, with a diet centered on rice, porridge, seafood, and fruits.

A second follow-up was successfully performed during lactation on 6 Han, 4 Li, and 8 Uyghur mother-infant dyads, with fecal samples collected from both mothers and infants (Figure 1B). A total of 110 samples were collected. The height (length), weight, and gender of the infant were documented, along with detailed information on maternal dietary patterns, daily living conditions, and infant feeding practices. Furthermore, the following information was also recorded in detail: (i) whether the mother had used antibiotics, taken probiotics, and a probiotic product containing live beneficial microbiota during pregnancy; (ii) vaginal delivery or cesarean delivery; (iii) whether all infants had been breastfeeding from birth until 6 months of age. Simultaneously, the woman and her family members were informed of the sampling purpose at the time of collection, and they agreed and signed the family members’ notification page and informed consent. During the sampling process, the sampler wore sterile gloves, scooped up at least 1 g of feces with a sampling spoon, and put it into a fecal collection tube. Moreover, feces and urine were kept separate. The collected samples were put into a − 20 °C vehicle refrigerator and transported to the laboratory immediately. Before DNA extraction, the samples were stored at −80 °C.

### DNA extraction

Total DNA was extracted from maternal and infant stool samples using the TIANamp Stool DNA Kit (Tiangen Biotech, China). Approximately 220 mg of feces were homogenized in a 2 mL centrifuge tube with 500 μL of buffer SA, 100 μL of buffer SC, 15 μL of Proteinase K (20 mg/mL), and 0.25 g of 1 mm grinding beads. The mixture was vortexed intermittently for 1 min to ensure thorough suspension, then incubated at 70 °C for 15 min. After incubation, the sample was centrifuged at 12,000 rpm (~13,400 × g) for 3 min, and the supernatant was transferred to a new tube. Subsequently, 10 μL of RNase A (10 mg/mL) was added, followed by incubation at room temperature for 5 min. Then, 200 μL of buffer SH was added, and the tube was placed on ice for 5 min before centrifugation at 12,000 rpm (~13,400 × g) for 5 min. The resulting supernatant was transferred to a fresh 1.5 mL tube, and an equal volume of buffer GFA was added. This mixture was loaded onto an adsorption column CR2, and DNA was purified according to the manufacturer’s protocol. To maximize DNA yield, the eluted DNA was reapplied to the same column and centrifuged again at 12,000 rpm (~13,400 × g) for 2 min. DNA quality and concentration were assessed using a Nanodrop2000 spectrophotometer. Samples with an OD260/280 ratio between 1.7 and 1.9 were considered acceptable and were stored at −20 °C for subsequent analyses.

### Full-length 16S rRNA sequencing for gut microbiota and *groEL*/*tuf* gene sequencing for *Bifidobacterium* species

Total DNA was extracted from 110 fecal samples and sent to Shanghai Personal Biotechnology Co., Ltd. (Shanghai, China) for microbial high-throughput sequencing. PacBio Seque1 was used for full-length amplicon sequencing of the 16S rRNA gene. The forward primer was 27F: 5’-AGAGTTTGATCMTGGCTCAG-3′, and the reverse primer was 1492R: 5′- ACCTTGTTACTT-3′. The PCR amplification parameters are as follows: predegeneration at 98 °C for 2 min, followed by 30 cycles, where 1 cycle consisted of denaturation at 98 °C for 15 s, annealing at 55 °C for 30 s, extension at 72 °C for 30 s, and a final extension at 72 °C for 5 min. Additionally, the *groEL* gene (Bif-*groEL*-F: 5’-TCCGATTACGAYCGYGAGAAGCT-3′, Bif-*groEL*-R: 5’-CSGCYTCGGTSGTCAGGAACAG-3′) and the *tuf* gene (*tuf*-F: 5′- GTCCGTGACCTCCTCGAC-3′, *tuf*-R: 5’-GTGGAAGGTCTCGATGGAG-3′) with good amplification ability for *Bifidobacterium* were selected, and the extracted fecal DNA was sequenced using the MiSeqPE300 and MiSeqPE250 platforms, respectively (Hu et al., 2017; Yang et al., 2021). The amplification procedures for *the groEL* gene included a pre-denaturation step at 95 °C for 5 min, followed by 35 cycles consisting of denaturation at 95 °C for 30 s, annealing at 58 °C for 30 s, and extension at 72 °C for 1 min, and a final extra extension at 72 °C for 7 min. For the *tuf* gene, the amplification protocol consisted of an initial denaturation at 94 °C for 5 min, followed by 35 cycles of denaturation at 94 °C for 35 s, annealing at 60 °C for 35 s, extension at 72 °C for 40 s, and a final extension at 72 °C for 7 min.

The PCR amplification system was 25 μL, including 5 × reaction buffer 5 μL, 5 × GC buffer 5 μL, dNTP (2.5 mM) 2 μL, Forward primer (10 μM) 1 μL, Reverse primer (10 μM) 1 μL, DNA Template 2 μL, double distilled water (ddH_2_O) 8.75 μL, Q5 DNA Polymerase 0.25 μL. The 25 μL PCR products were mixed with Vazyme VAHTSTM DNA Clean Beads at 0.8 times the volume to purify and recycle the amplified products. Then, the Quant-iT PicoGreen dsDNA Assay Kit was used to quantify the fluorescence of the PCR-amplified products. According to the fluorescence quantification results, all samples were mixed in proportions consistent with the sequencing requirements of each sample. Illumina’s TruSeq Nano DNA LT Library Prep Kit was used to prepare a sequencing library, and a sequencing connector containing a library-specific tag (i.e., Index sequence) was added to the 5′ end of the sequence. Finally, the qualified computer sequencing libraries were diluted in a gradient, mixed in proportion to the required sequencing volume, and denatured into a single strand by NaOH for high-throughput sequencing.

### Bioinformatics and statistical analysis

QIIME2-2022.8, USEARCH v11.0, VSEARCH v2.15.2, and R 4.2.2 were used for quality filtering and bioinformatics analysis of the original sequencing data. The sequencing quality was first evaluated using FastQC v0.12.1. Individual reports were merged into a single one with MultiQC v1.19. The bioinformation analysis process follows the EasyAmplicon v1.13 (Liu et al., 2020). The pipeline included quality control and de-duplication of sequences using the --fastx_filter and --derep_fulllength subcommands in VSEARCH v2.15.2, respectively. Non-redundant sequences were then denoised into amplicon sequence variants (ASVs), and a feature table was generated using QIIME2. Representative sequences from the 16S rRNA gene dataset were classified against the SILVA database (release 138.1). For the *groEL* and *tuf* gene datasets, characteristic sequences were classified at the *Bifidobacterium* species level using the chaperone protein sequence database (CPNDB)^1^ and the NCBI database.

### Data visualization

Data visualization was performed using the ggplot2 v3.3.2 package, and diversity analysis was calculated by QIIME2 and the vegan v2.6–4 package. Differences between groups were assessed with Wilcoxon rank-sum tests. The produced *p*-values were adjusted for multiple testing using the Benjamini-Hochberg false-discovery rate (of 5%, FDR). Correction for multiple testing followed the Benjamini-Hochberg procedure (p.adjust() function of the R package stats 4.2.2, parameter method = BH). The FDR < 0.05 was considered statistically significant. For more than two groups, the Kruskal–Wallis test with *post-hoc* Dunn tests was used. Bray–Curtis distances were computed as beta diversity and used for principal component analysis (PCA). We calculated the average abundance of dominant ASVs in each ethnic group. A Venn diagram was drawn using Evenn^2^ (Yang et al., 2024). Transmission mode (TM) scores were calculated according to the method described by Moeller et al. (2018). Obligate anaerobes tended to be transmitted vertically (TM score > 1), whereas obligate aerobes were transmitted horizontally (TM score < 1). The heatmap was generated using the pheatmap v1.0.12 package in R software. Microbial composition was mapped using the R package amplicon v1.18.2. Before phylogenetic analysis, 26 *Bifidobacterium* ASVs were aligned using MUSCLE v5.1. The resulting alignment was used as the input to construct a maximum-likelihood phylogenetic tree in IQ-TREE with automatic model selection. Branch support was assessed using ultrafast bootstrap approximation with 1,000 replicates (Hoang et al., 2018) in IQ-TREE 2 (Lanfear et al., 2020). The output tree file was imported into the Interactive Tree Of Life (iTOL) for visual refinement (Letunic and Bork, 2016). Furthermore, the distribution profiles of these 26 ASVs across 54 mother-infant pairs were integrated into iTOL and visualized as a heatmap matrix.

### Recovery of *Bifidobacterium* isolates and multilocus sequence typing (MLST) analysis

The fecal samples were serially diluted with sterile saline supplemented with 0.5% L-cysteine hydrochloride (Sigma) after thorough suspension by vortexing. The dilution suspension (10^−3^, 10^−4^, 10^−5^) were then plated onto de Man-Rogosa-Sharpe (MRS) medium supplemented with 0.05% (w/v) L-cysteine hydrochloride and mupirocin (50 mg/L) and *Bifidobacterium* selective agar {MAN agar [Wilkins-Chalgren agar supplemented with soya peptone (5 g/L), L-cysteine (5 g/L), Tween 80 (1 mL/L), mupirocin (100 mg/L), and glacial acetic acid (1 mL/L)]; Vlková et al., 2015}. Each gradient dilution was spread on two plates and cultured at 37 °C for 48–72 h under anaerobic conditions. The morphology of the bacteria was observed under a microscope. From each plate, 10–15 colonies were selected, and 40–50 colonies were selected from each sample according to the size and color of the bacteria. After purification at least three times, the pure cultures were stored in MRS broth containing 25% glycerol at −80 °C. DNA was extracted from 1.0 mL of bacterial culture grown in mMRS + 0.5% (w/v) L-cysteine hydrochloride and mupirocin (50 mg/L) using the FastPure Bacteria DNA Isolation Mini Kit (Vazyme) according to the instructions received from the manufacturer with slight modifications. Specifically, 1 mL of bacterial culture solution was centrifuged at 12,000 rpm for 10 min at 4 °C. Discard the supernatant liquid. The pellets were resuspended in 200 μL of Tris-EDTA buffer and treated with 10 μL of lysozyme (50 mg/mL) and 4 μL of DNase-free RNase (20 mg/mL) for 30 min at 37 °C. Twenty-five milligrams of glass beads (10 μm) were added to the solution and treated with three bead-beating steps in a FastPrep instrument (MP Biomedicals, Irvine, CA, United States) at 5.5 movements per second for 1 min. After the instantaneous centrifugation, the supernatants were collected and treated with 20 μL of proteinase K for 20 min at 56 °C. 250 μL of GB buffer was added, and the samples were incubated at 70 °C for 10 min; then 250 μL of ethanol was added. DNA was further purified using FastPure gDNA Mini columns (Vazyme) following the manufacturer’s instructions. Identification of the strains at the species level was carried out by PCR sequencing of the *groEL* gene (Hu et al., 2017; Yang et al., 2021).

Seven housekeeping genes (*clpC*, *fusA*, *gyrB*, *ileS*, *purF*, *rplB*, *rpoB*) encoding proteins were chosen for analysis (Supplementary Table S1; Delétoile et al., 2010; Makino et al., 2011; Yanokura et al., 2015; Zhang et al., 2015; Syst. Appl. Microbiol., n.d). PCR products were verified by electrophoresis in 1.2% (w/v) agarose gels and sequenced by GENEWIZ (Jiangsu, China). Seven housekeeping gene sequence datasets from strains were edited and aligned using Chromas v2.6.6 and then downloaded to the MLST database established in BioNumerics v8.0 (Applied-Maths, Sint-Martens-Latem, Belgium). Each distinct gene sequence was assigned an allele number, and each unique combination of seven allele numbers was assigned a sequence type (ST). The minimum spanning tree (MST) analysis was performed using Prim’s algorithm embedded in BioNumerics software based on isolation sources and regions. We created a dendrogram using the multiscale setting for comparisons and the unweighted pair group method with arithmetic mean (UPGMA) for clustering.

### Construction of mock *Bifidobacterium* communities

Three mock communities (MCs) were constructed to evaluate the *groEL* and *tuf* gene-based culture-independent identification method for *Bifidobacterium* used in this study; 6 bacterial strains belonging to 5 different *Bifidobacterium* species, namely *B. longum* subsp. *longum*, *B. breve*, *B. bifidum*, *B. adolescentis*, and *B. pseudocatenulatum*, which were mixed at different cell concentrations. All the bifidobacterial strains were grown in MRS medium with 0.05% (wt/vol) L-cysteine hydrochloride and 0.01% (wt/vol) mupirocin under anaerobic conditions at 37 °C for 48–72 h. The purity of bacterial cultures was checked by Gram staining. The identities of the *Bifidobacterium* strains underlined in Supplementary Table S2 were confirmed at the species level by sequencing the *groEL* gene. The cell concentration of each culture was checked by measuring the optical density at 600 nm. Cells were pelleted by centrifugation at 3,000 × g for 10 min at 4 °C and washed with sterile deionized water. After a second centrifugation at 3,000 × g, cells were resuspended in sterile water to obtain a concentration of 10^9^ cells/mL. Mock communities were then created by mixing the different strains at the desired concentration ratio. Mock community mixture was collected for DNA extraction. Subsequently, the extracted DNA was subjected to sequencing targeting the *groEL* and the *tuf* genes.

## Results

### Overview of the mother-infant cohort

We conducted a longitudinal characteristic analysis of the gut microbiota of 110 samples from 54 mother-infant pairs from three different ethnic groups in China (two stages of lactation, the neonatal period, and the 6-month-old infant period), including 14 pairs of Han ethnicity, 14 pairs of Li ethnicity, and eight pairs of Uyghur ethnicity (Figure 1B). The demographic data indicated that 58.33% of the volunteers lived in rural areas, while the remaining 41.67% lived in urban areas. None of the mothers took any antibiotics or probiotics during the sampling period. At 7 days postpartum, 48.15% of mothers had normal weight (Han: 18.52%, Li: 22.22%, Uyghur: 7.41%), 37.04% were overweight (Han: 14.81%, Li: 11.11%, Uyghur: 11.11%), and 14.81% were obese (Han: 3.71%, Uyghur: 11.11%). At 6 months postpartum, 3.70% were underweight (all Li), 51.85% had normal weight (Han: 14.81%, Li: 29.63%, Uyghur: 7.41%), 40.75% were overweight (Han: 22.22%, Uyghur: 18.52%), and 3.70% were obese (all Uyghur). Statistical analysis of delivery modes showed that in this cohort, 61.11% of the infants were delivered vaginally and 38.89% were delivered by cesarean. Furthermore, all newborns in this cohort were breastfed within 7 days after birth. By 6 months of age, they all followed a mixed feeding pattern, consuming both breast milk and complementary foods (Table 1). In this neonatal cohort, male infants had an average birth weight of 3.28 ± 0.34 kg and a body length of 49.95 ± 5.55 cm, while female infants had an average birth weight of 3.15 ± 0.26 kg and a body length of 47.57 ± 3.05 cm. All neonatal growth indicators conformed to the Growth Standards for Children under 7 Years Old (WS/T 423–2022) issued by the National Health Commission of the People’s Republic of China. At 6 months of age, only 2 of 7 female infants and 6 of 21 male infants presented slight deviations in body length and weight relative to the national reference criteria. Importantly, the nine infants did not meet the diagnostic thresholds for low weight, wasting, or stunted growth as stipulated in WS/T 423–2022. Overall, all infants enrolled in this study maintained a normal, healthy nutritional status defined by national standards.

**Table 1:** Demographic characteristics of the mothers and infants in the study population.

Characteristics and demographic data	Values	
Lifestyle	Mother	
Rural	21 (58.33%)	
Urban	15 (41.67%)	
BMI	Mother-delivery	Mother-postpartum 6 months
Underweight (< 18.5)	0 (0.00%)	1 (3.70%)
Normal (18.5–24.9)	13 (48.15%)	14 (51.85%)
Overweight (25.0–29.9)	10 (37.04%)	11 (40.75%)
Obesity (≥ 30)	4 (14.81%)	1 (3.7%)
Delivery mode	Infant	
Vaginally	22 (61.11%)	
Cesarean	14 (38.89%)	
Infant weight (kg)	Infant-7 days	Infant-6 months old
Male	3.28 ± 0.34	6.98 ± 1.58
Female	3.15 ± 0.26	5.89 ± 1.27
Infant length (cm)	Infant-7 days	Infant-6 months old
Male	49.95 ± 5.55	64.76 ± 6.19
Female	47.57 ± 3.05	62.57 ± 6.11
Feeding methods	Infant-7 days	Infant-6 months old
Breastmilk	28 (100%)	0
Mixed feeding	0	28 (100%)

### Characterization of the mother-infant pairs’ intestinal microbial diversity and composition by means of 16S rRNA-based microbial profiling

**Figure 2:**
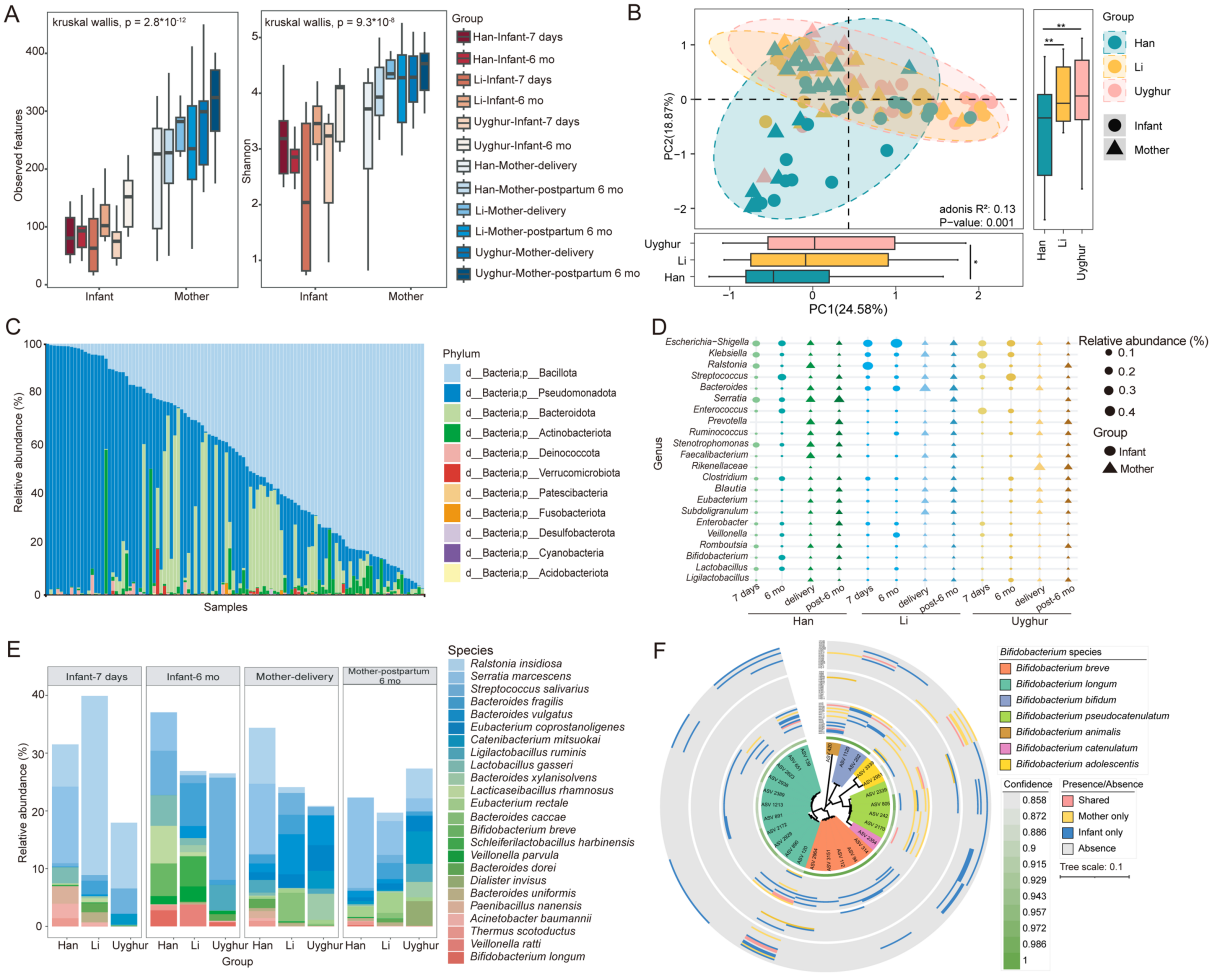
Overview of the gut microbiota diversity and composition of mother-infant cohorts based on the 16S rRNA full-length gene. **(A)** Observed features and Shannon *α*-diversity indexes among the three ethnic groups of mothers and infants between the two sampling time points. The Kruskal–Wallis test was used for a significance test. **(B)** PCA plot of mothers and infants across three ethnic groups based on Bray-Curtis distance. **(C)** The relative abundance of each phylum in each sample. **(D)** The average relative abundance of the 23 most prevalent genera (*y*-axis) with an average relative abundance >1% in each group (*x*-axis) is plotted for infants and mothers at delivery and 6 months. **(E)** Columnar accumulation map of relative abundance for discriminating species (rows) with an average relative abundance >0.3%. **(F)** Maximum likelihood tree of 26 ASVs belonging to *Bifidobacterium* member based on the 16S rRNA gene and their co-occurrence in mother-infant dyads.

Based on 16S rRNA full-length high-throughput sequencing, a total of 887,543 reads were generated from 110 fecal samples. These reads were denoised using DADA2 and classified as ASVs (Amplicon sequence variants), generating a total of 3,358 ASVs. Microbial richness was estimated by observed features and the Chao1 index, and microbial diversity was assessed using the Shannon and Simpson indices. The alpha diversity analysis revealed that microbial richness indices were significantly higher in 6-month-old infants relative to neonates. Moreover, the intestinal microbial richness of 6-month-old Uyghur infants was significantly higher than that of Li and Han infants (Figure 2A; Supplementary Table S3). For beta diversity, PCA based on Bray-Curtis distance showed clear separation of microbial community structure among mother-infant samples from different ethnic groups (R^2^ = 0.13, *p* = 0.001; Figure 2B). The boxplot results of PC1 and PC2 scores indicated that the Han group had lower PC1 and PC2 scores than the Li and Uyghur groups, with the Uyghur group displaying the highest scores. Significant differences in both PC1 and PC2 scores were further detected between the Han and Uyghur groups (*p* = 0.012*, 0.0042**).

Bacillota (45.09 ± 31.07%), Pseudomonadota (39.24 ± 35.75%), Bacteroidota (11.68 ± 18.99%), and Actinobacteria (3.05 ± 7.94%) were the four most abundant bacterial phyla in fecal samples (Figure 2C). Among all the fecal samples, there were 22 genera with an average relative abundance greater than 1% (Figure 2D). *Bacteroides* (7.09 ± 12.82%), *Serratia* (6.46 ± 16.05%), and *Prevotella* (5.80 ± 14.27%) were enriched in the mother group. *Escherichia_shigella* (21.03 ± 25.69%), *Klebsiella* (10.76 ± 21.45%), and *Streptococcus* (9.60 ± 17.96%) were the dominant microbiota in the infant gut. Several bacterial classes displayed a mean genus-level TM score significantly different from 1 (based on 95% confidence intervals): *Bacteroidia* (TM = 1.206), *Bifidobacterium* (TM = 1.150), while *Streptococcus* (TM = 0.959), *Ruminococcus* (TM = 0.872), *Staphylococcus* (TM = 0.729), and *Prevotella* (TM = 0.719; Supplementary Figure S1A). Additionally, *Escherichia* was detected in the three ethnic mother and infant groups. At species level, *Serratia marcescens* (5.99% ± 16.05%), *Ralstonia insidiosa* (2.92% ± 12.50%), *Bacteroides vulgatus* (2.61% ± 7.20%), *Eubacterium coprostanoligenes* (2.07% ± 3.38%), *Catenibacterium mitsuokai* (1.58% ± 3.63%) appeared to be predominant in the mother gut; while *Ralstonia insidiosa* (8.55% ± 22.70%), *Streptococcus salivarius* (5.44% ± 13.67%), *Serratia marcescens* (3.32% ± 10.36%), *Bacteroides fragilis* (1.78% ± 4.93%) and *Lactobacillus gasseri* (1.59% ± 6.20%) were more abundant in the infant group (Figure 2E).

Notably, across the three ethnic groups, the 6-month-old infant group exhibited a higher abundance of *Bifidobacterium* than the newborn group (5.95% vs. 0.07%). Only 57 out of the 110 maternal and infant samples (maternal group: 28/54; infant group: 29/56 volunteers) were found to have a prevalence of *Bifidobacterium*.

Based on the species annotation results from SLIVA 138.1, we further classified the bifidobacterial characteristic sequences with greater precision using comparative analyses from the National Center for Biotechnology Information (NCBI). Among the 3,358 ASVs, 26 ASVs belonged to *Bifidobacterium* (detection rate: 0.77%), including 7 *Bifidobacterium* species: *B. longum* (11 ASVs, average relative abundance: 0.478%), *B. breve* (5 ASVs, 0.606%), *B. pseudocatenulatum* (4 ASVs, 0.169%), *B. bifidum* (2 ASVs, 0.139%), *B. adolescentis* (2 ASVs, 0.016%), *B. animalis* (1 ASV, 0.044%) and *B. catenulatum* (1 ASV, 0.0089%), respectively (Figure 2F). *B. pseudocatenulatum* and *B. longum* group were more abundant in the Han mothers, while *B. animalis* was more abundant in the Uyghur and Li mothers. In the infant cohort of this study, *B. breve* (average relative abundance: 1.57%), *B. longum* (0.95%), and *B. bifidum* (0.33%) were relatively abundant. The relative abundance of *B. longum* was higher in Uyghur infants, and infants of the Han ethnic group were more abundant in *B. breve*, while *B. pseudocatenulatum* had the highest relative abundance in the Li group (Figures 2E,F). The results of the ML tree constructed from the characteristic ASVs of *Bifidobacterium* indicated that the sequences belonging to the same *Bifidobacterium* species were clustered on the same evolutionary clade. Heatmap analysis further revealed a consistent co-occurrence of identical *Bifidobacterium* characteristic sequences in matched mother-infant dyads. For example, ASV 120 of the *B. longum* was shared in the mother-infant pairs of wwc29, wwc4, HTb17, and HTb19, and ASV139 was shared in wwc10. Similarly, it was observed that the ASV 202 belonging to the *B. bifidum* was co-occurrence in wwc29 (Figure 2F). Overall, among mother-infant pairs with continued breastfeeding at 6 months postpartum, the co-occurrence rate of *Bifidobacterium* ASVs was higher than that during the neonatal period.

### Infants’ microbiota is more similar to their mother’s

**Figure 3:**
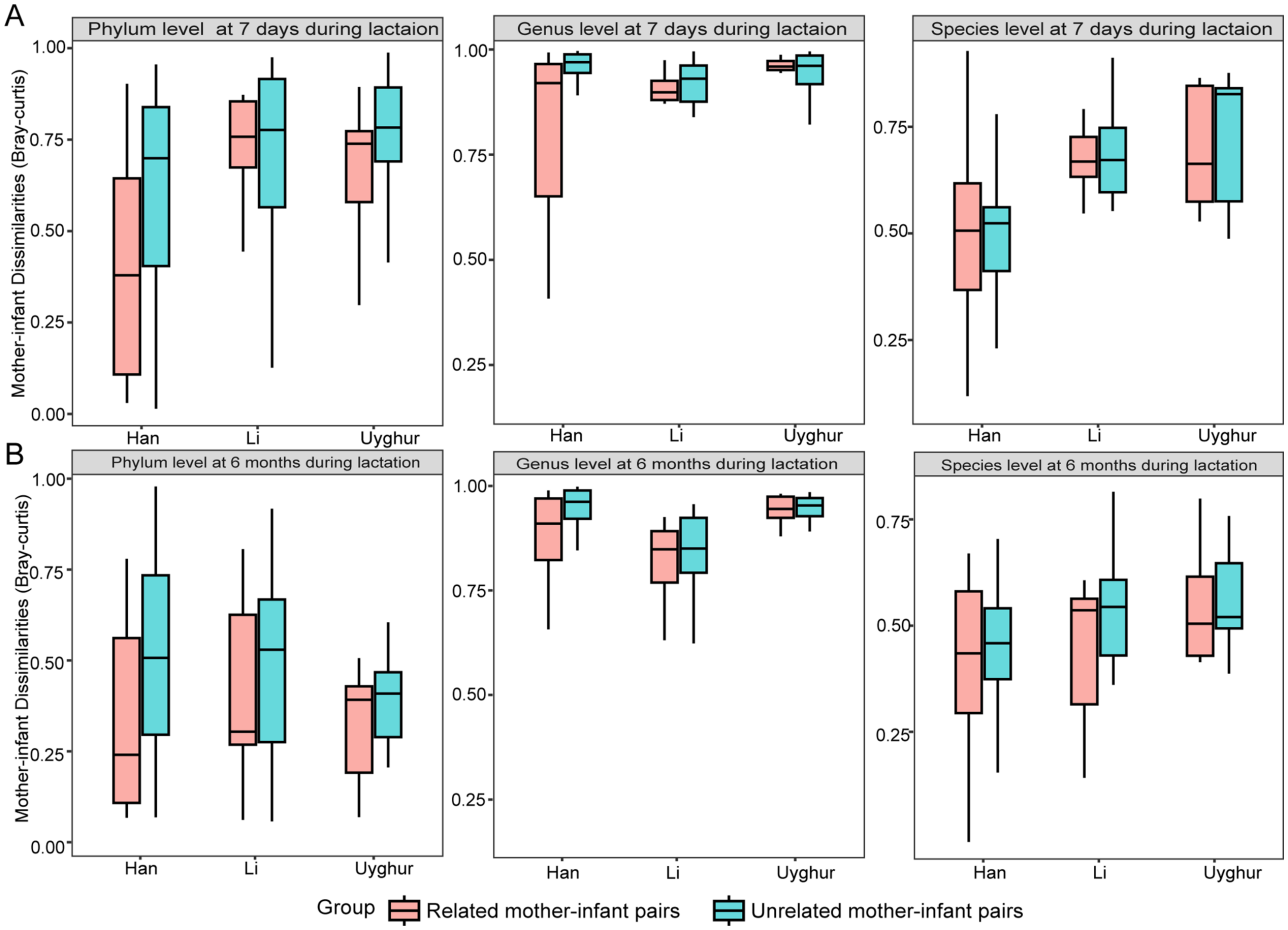
Box plots of inter-ethnic microbial dissimilarity across ethnic groups during two lactation stages: **(A)** The initial 7 days postpartum period and **(B)** the 6-month, calculated using Bray-Curtis distance.

We calculated the average number of genera, species, and ASVs shared by mother-infant pairs in the fecal samples (Table 2). The Han ethnic group shared the most microbiota, followed by the Uyghur and Li. Furthermore, under continuous breastfeeding, the percentage of shared microbes between the mother and infant microbiota at 6 months of lactation was higher than at the initial time. As expected, the fraction of shared genus was much higher than the fraction of shared species. Similarity between the microbial profiles of Han, Li, and Uyghur mother-infant pairs was quantified using the Bray-Curtis distance-based approach. Bray-Curtis dissimilarities were computed for each mother-infant pair at the phylum, genus, and species levels, respectively. As shown in Figure 3, when comparing ethnic groups, maternal–infant pairs within the same ethnic group were closer together at both 7 days and 6 months postpartum, regardless of the microbial resolution approach. The dissimilarity index of the mother-infant pair decreased with the growth of infants; that is, during breastfeeding, the similarity of intestinal microbes between mothers and infants increased (Figures 3A,B).

**Table 2:** The sharing rate of the mother-infant dyad cohorts at different classification levels based on 16S rRNA gene.

Genus-level	Han-7 days	Han-6 mo	Li-7 days	Li-6 mo	Uyghur-7 days	Uyghur-6 mo
Mother Only (%)	49.65	57.84	70.73	60.74	72.90	56.72
Shared (%)	22.26	23.51	13.82	19.47	16.16	22.41
Infant Only (%)	28.09	18.65	15.45	19.78	10.94	20.87
Species-level						
Mother Only (%)	52.63	59.66	72.41	61.14	75.07	50.41
Shared (%)	14.04	15.55	8.71	14.20	10.92	15.31
Infant Only (%)	33.33	24.79	18.88	24.65	14.01	34.29
ASV-level						
Mother Only (%)	56.48	63.35	77.65	61.70	82.42	52.88
Shared (%)	8.99	7.54	3.12	6.07	3.94	6.68
Infant Only (%)	34.52	29.11	19.23	32.22	13.64	40.44

### Evaluation of the bifidobacterial diversity and composition at the species level profiled by *groEL* and *tuf* genes

**Figure 4:**
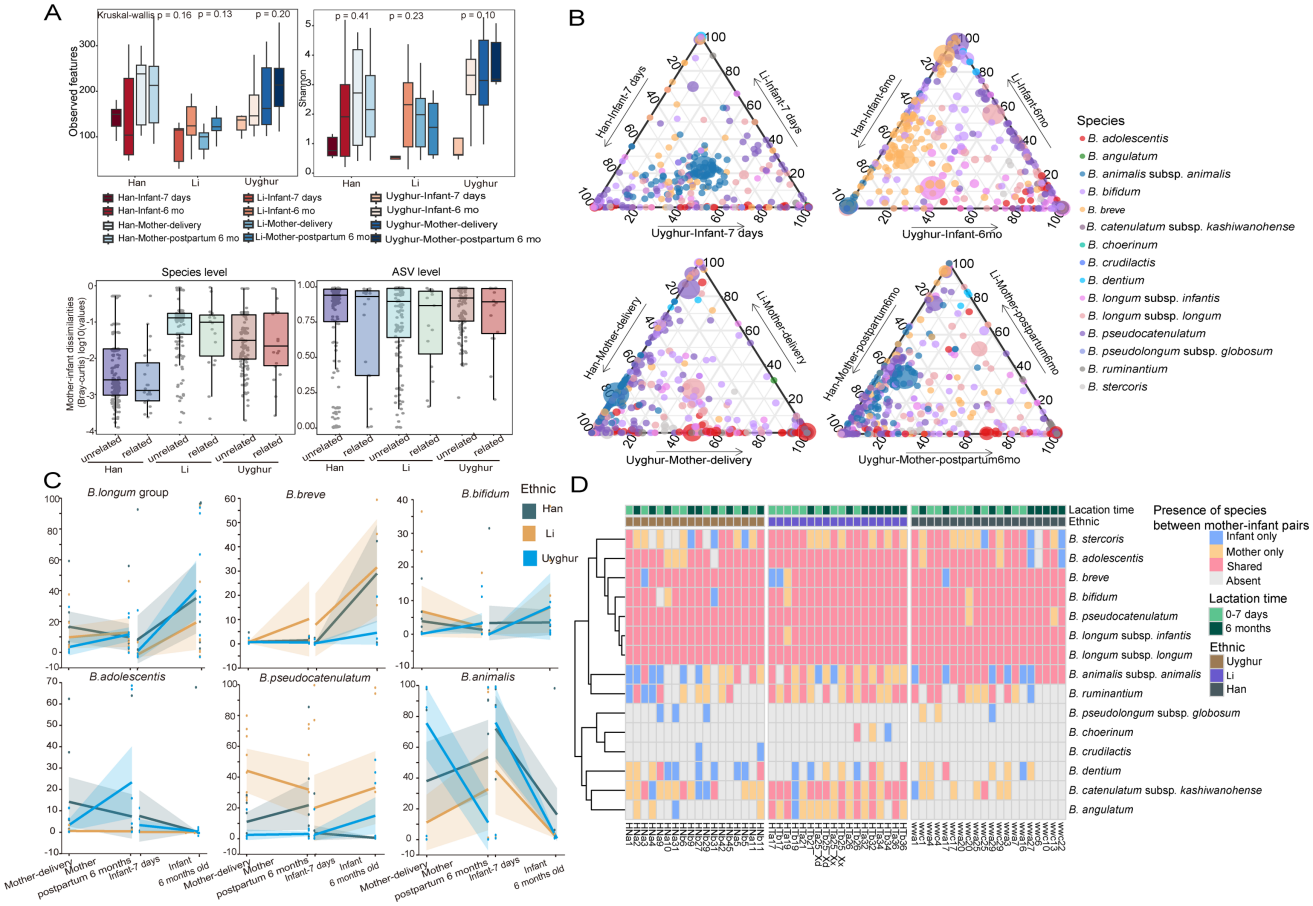
*Bifidobacterium* diversity and composition profiles of mother-infant pairs based on the *groEL* gene. **(A)** The box plot of observed features and Shannon index at the species level. Bray–Curtis dissimilarity index across the three ethnic groups at *Bifidobacterium* species and ASV level. ^*^p-value < 0.05, ^**^p-value < 0.01, ^***^p-value < 0.001. **(B)** Ternary map of species composition of *Bifidobacterium* based on the *groEL* gene. **(C)** The dynamic changes of *B. longum* group, *B. breve*, *B. bifidum*, *B. aolescentis*, *B. pseudocatenulatum*, and *B. animalis* group in maternal and infant of different ethnic groups at 0–7 days and 6 months, respectively. The figure shows the ratio of differences in the relative abundance of bifidobacterial species over 95% confidence intervals. **(D)** Heatmap of shared *Bifidobacterium* species of 54 mother-infant pairs. Pink: detected in related mother-infant pairs; yellow: only detected in the mother; blue: only detected in the infant; gray: not detected at all.

At the bifidobacterial-ASV level, results for bifidobacterial-specific genes showed an increase in alpha diversity (observed features and Shannon index) with infant age. However, they were still lower at 6 months compared to the diversity of the mothers’ gut microbiota (Figure 4A; Supplementary Table S5). Non-metric multidimensional scaling (NMDS) analysis of *groEL* and *tuf* genes indicated that there was a difference in the community structure of *Bifidobacterium* in mother-infant dyad cohorts of different ethnicity (*groEL*: R^2^ = 0.11, *p* = 0.003; *tuf*: R^2^ = 0.16, *p* = 0.001, Bray-Curtis based PERMANOVA; Supplementary Figure S2B). Based on the dissimilarity index calculated by the Bray-Curtis distance (measured by the ASVs of each *Bifidobacterium* species identified by *groEL* and *tuf* genes), the microbiota of *Bifidobacterium* was more similar between related mother-infant pairs (Figure 4A). *groEL* profiling of all 110 samples generated a total of 4,528,099 reads, which were grouped into 1,623 ASVs of identical sequences. Taxonomic annotation showed that 743 ASVs were assigned to at least 10 members of *Bifidobacterium* and 5 subspecies. As for the *tuf* gene, a total of 5,973,580 high-quality reads were obtained from 97 samples (the remaining 13 samples failed to complete sequencing), which were then classified as 1,337 ASVs. 99.85% of the sequencing results were identified as *Bifidobacterium*, classification notes revealed that 827 of the ASVs belonged to at least nine members of *Bifidobacterium* and 8 subspecies through *tuf*-profiling analysis (Figure 4B).

Results showed that the *groEL* and the *tuf* gene encompass members of the species *B. animalis* group, *B. pseudocatenulatum*, *B. longum* group, *B. adolescentis*, and *B. catenulatum* subsp. *kashiwanohense*, *B. breve*, *B. bifidum*, *B. angulatum*, *B. dentium*, and *B. pseudolongum* group. Additionally, *B. colobi*, *B. dolichotidis*, *B. minimum,* and *B. pullorum* subsp. *saeculare* has been detected only with the *tuf* gene. Notably, the *tuf* gene identified a total of four subspecies of the *B. longum* group (*B. longum* subsp. *infantis*, *B. longum* subsp. *longum, B. longum* subsp. *suis*, and *B. longum* subsp. *suillum*), while the *groEL* gene could identify only *B. longum* subsp. *infantis*, *and B. longum* subsp. *longum*. The *groEL* and *tuf* genes both indicated that the maternal gut has more *B. pseudocatenulatum* and *B. adolescentis*, especially in the maternal group of Li. *B. animalis* group was the most dominant species among gut bifidobacteria in the neonatal period across the three ethnic groups, followed by *B. longum* group. From 7 days to 6 months, the relative abundance of the *B. animalis* group decreased, replaced by an increase in the relative abundance of the *B. longum* group, *B. breve,* and *B. bifidum* (Figure 4C; Supplementary Table S6).

### The shared distribution of *Bifidobacterium* species and the dynamic changes between related mother-infant pairs

**Figure 5:**
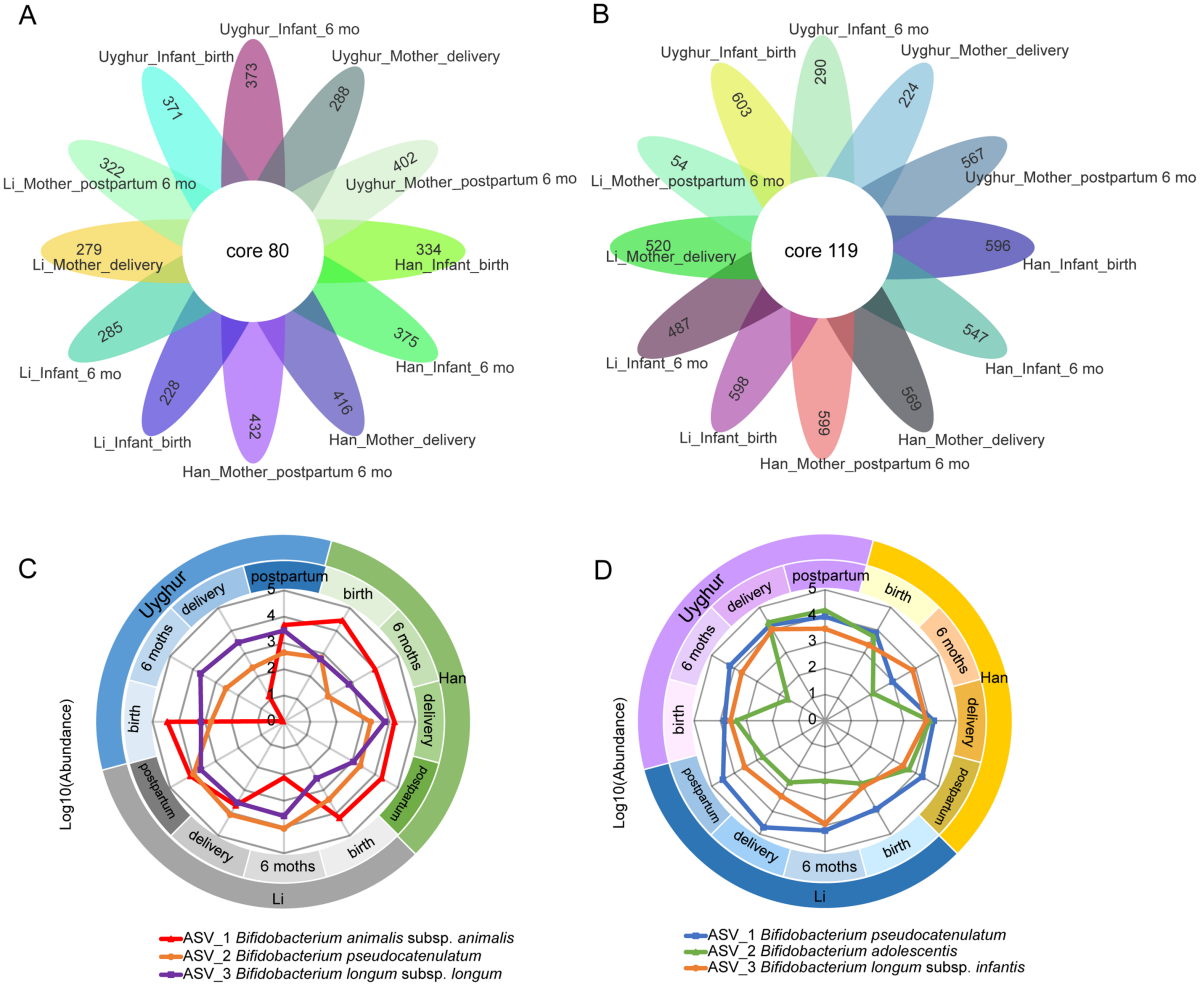
Distribution of population type-specific amplified sequence variants (ASVs) of *Bifidobacterium*. Flower plots displayed the number of shared and unique ASVs in 12 groups, including three ethnic groups at two lactation time points for mothers and infants based on *groEL* **(A)** and *tuf* gene **(C)**. The radar charts show the distribution of the three most abundant ASVs concentrated in the *groEL* **(B)** and *tuf* **(D)** gene dataset. The three ASVs with the highest abundance were also common across all groups.

Based on the *groEL* gene dataset, 80 ASVs belonging to 10 *Bifidobacterium* species were shared across the three ethnic infant groups and maternal groups, excluding *B. dentium* (Figure 5A; Supplementary Table S7). Additionally, unique detections included *B. pseudolongum* subsp. *globosum* in the Han pairs cohort, *B. angulatum* in both Li and Uyghur pairs, *B. choerinum* exclusively in Uyghur pairs, and *B. crudilactis* solely in 6-month-old Li infants. Generally, *B. longum* subsp. *longum* had the highest sharing rate among the three ethnic mother-infant pairs (100%), followed by the *B. longum* subsp. *infantis* (98.21%) and *B. pseudocatenulatum* (91.07%; Supplementary Table S7).

In the *tuf* gene dataset, *B. longum* subsp. *infantis* and *B. pseudocatenulatum* were universally shared by mother-infant pairs, while *B. longum* subsp. *longum* had only 37.21% sharing rate. The *tuf* gene-based classification identified 17 species or subspecies of *Bifidobacterium*, of which 14 species (including subspecies) were observed in the three ethnic mother-infant groups. These comprised *B. animalis* subsp. *lactis*, *B. longum* subsp. *infantis*, *B. longum* subsp. *longum*, *B. longum* subsp. s*uis*, and *B. longum* subsp. *suillum*, *B. adolescentis*, *B. pseudocatenulatum*, *B. breve*, *B. bifidum*, *B. catenulatum* subsp. *kashiwanohense*, *B. dentium*, *B. angulatum*, *B. colobi*, and *B. pseudolongum* subsp. *pseudolongum*. Across the three ethnic infant and maternal groups, 119 ASVs belonging to 10 species or subspecies were shared, including *B. animalis* subsp. *lactis*, *B. longum* subsp. *infantis*, *B. longum* subsp. *longum*, *B. adolescentis*, *B. pseudocatenulatum*, *B. breve*, *B. bifidum*, *B. catenulatum* subsp. *kashiwanohense*, *B. dentium*, and *B. colobi* (Figure 5B; Supplementary Table S8). Furthermore, *B. dolichotidis* and *B. minimum* were detected specifically in Han and Li infants, while *B. pullorum* subsp. *saeculare* was uniquely present in 6-month-old Li and Uyghur infants.

The results of the *groEL* and *tuf*-profiling analysis showed that the relative abundance of selected infant-type HRB (*B. longum* group, *B. breve*, and *B. bifidum*) increased with the growth of infants in the three ethnic groups, while the relative abundance of certain adult-type HRB (*B. adolescentis*, *B. pseudocatenulatum*, and *B. animalis* group) was higher in maternal samples (Figure 4D; Supplementary Figure S2D). To explore the ecological significance of microdiversity, we analyzed the distribution preferences of ASVs with the highest relative abundance in the samples and that were common across all groups. Analysis of ASV levels in the bifidobacterial-specific gene sets showed that infant-type HRB (ASV_1 *B. longum* subsp. *longum* in the *groEL* dataset and ASV_1 *B. longum* subsp. *infantis* in the *tuf* dataset) increased with the development of the infant (Figures 5C,D). Adult-type HRB was more abundant in the mother group, and there was little difference in abundance between the two lactation periods.

### Classification of the mock *Bifidobacterium* communities based on the *groEL* and *tuf* genes

We compared the resolution of the *groEL* and *tuf* gene amplicon sequencing for the taxonomic classification of bifidobacteria. The species added to the mock community based on the *groEL* gene amplicon were accurately identified, while the *tuf* dataset misidentified the *B. longum* subsp. *longum* strain FMBL B24239 as subsp. *infantis*. Furthermore, the unassigned proportion of the three mock communities (M1, M2, M3) in the *tuf* gene dataset was higher than in the *groEL* gene dataset. The concentrations of strains added in this study were 9.5, 9.7, 9.6, 7.5, and 9.5 log (CFU/mL) for *B. longum* subsp. *longum*, *B. breve*, *B. adolescentis*, *B. bifidum* and *B. pseudocatenulatum*. With equal volume addition of strains in M1 community, the *groEL* gene results showed that the content of *B. breve* was underestimated, and the relative abundance of other species was more accurately expressed, while the content of *B. breve* and *B. bifidum* in the *tuf* gene data set was overestimated, and the other species was underestimated. The M2 community added *B. bifidum* at twice the volume of M1; the amplification results for the marker genes showed that the relative abundance of *B. bifidum* increased by double compared with M1. Mock community M3 increased the volume of *B. pseudocatenulatum* twice based on M1, and the results showed that the *tuf* gene more accurately reflected the change of the relative content of *B. pseudocatenulatum* in the mock community than the *groEL* gene. Through mock communities, our results showed that the *groEL* gene can more accurately reflect the composition of *Bifidobacterium* in the community than the *tuf* gene (Supplementary Table S9).

### Culture-dependent analysis of *Bifidobacterium* strains and MLST profiles of three dominant *Bifidobacterium* lineages

**Figure 6:**
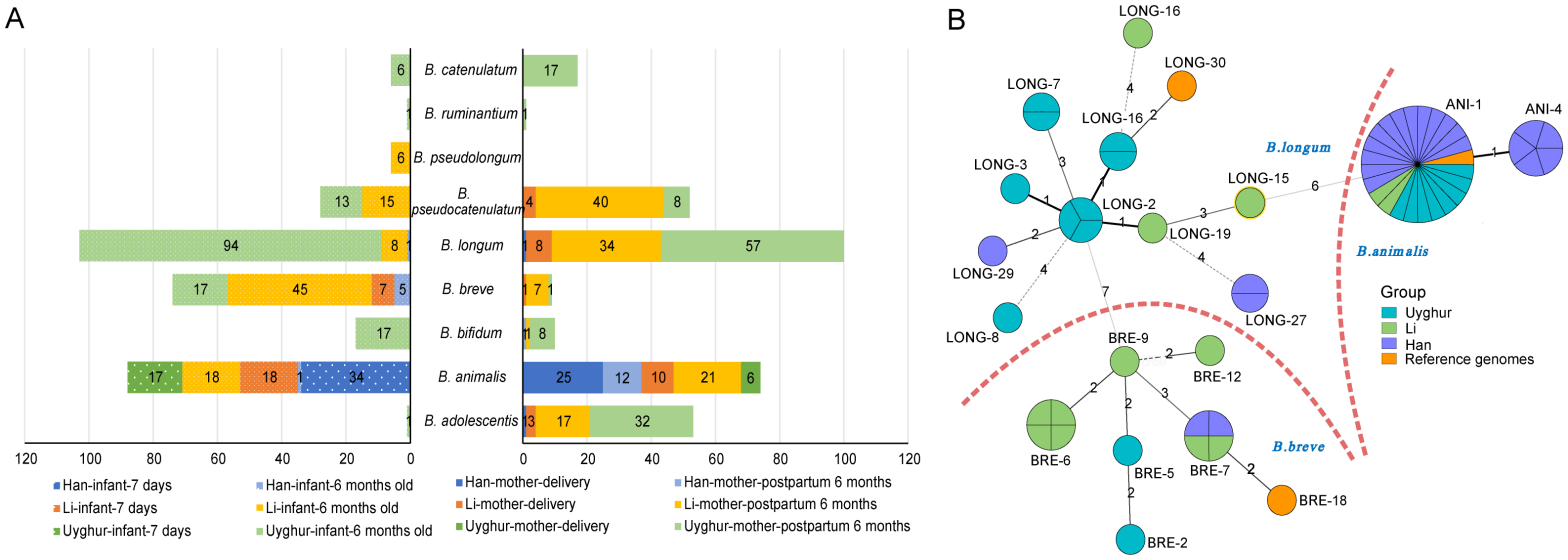
Analysis of *Bifidobacterium* species isolates and comparison of MLST profiles of *Bifidobacterium longum*, *Bifidobacterium breve*, and *Bifidobacterium animalis*. **(A)** Bar plot of *Bifidobacterium* strains isolated from each mother-infant pair of the three ethnic groups. The different colors represent different ethnic groups, and the left side of the bar chart is the infant group, and the right side is the mother group. The numbers in the bar chart represent the number of *Bifidobacterium* species isolated from the relevant cohort. **(B)** Minimum spanning tree of 55 *Bifidobacterium* strains based on MLST profiles according to the isolation source. Each filled circle corresponds to an ST. The color of the circle represents the source of isolation. Blue, Uyghur; Green, Li; Dark blue, Han.

We isolated 640 strains of *Bifidobacterium* belonging to 9 species from 110 fecal samples of 54 mother-infant pairs across three ethnic groups, including 203 strains of *B. longum* subsp. *longum*, 162 strains of *B. animalis* subsp*. lactis*, 83 strains of *B. breve*, 80 strains of *B. pseudocatenulatum*, 54 strains of *B. adolescentis*, 27 strains of *B. bifidum*, 23 strains of *B. catenulatum*, 6 strains of *B. pseudolongum*, and 2 strains of *B. ruminantium*. Among them, 316 and 324 strains were isolated from the mother and infant group, respectively. *B. longum* subsp. *longum* and *B. animalis* were detected at relatively high frequencies in both maternal and infant feces. *B. breve* predominated in the infant feces, and *B. adolescentis* was more abundant in the mother group. A total of 81 *Bifidobacterium* strains were divided into 5 species for the Han group, 263 strains into 7 species for Li, and 296 strains into 8 species for the Uyghur group. *B. pseudolongum* was only detected in 6-month-old infants of Li, whereas *B. ruminantium* and *B. catenulatum* were found in both 6-month-old infants and their corresponding mothers belonging to the Uyghur ethnic group (Figure 6A).

**Figure 7:**
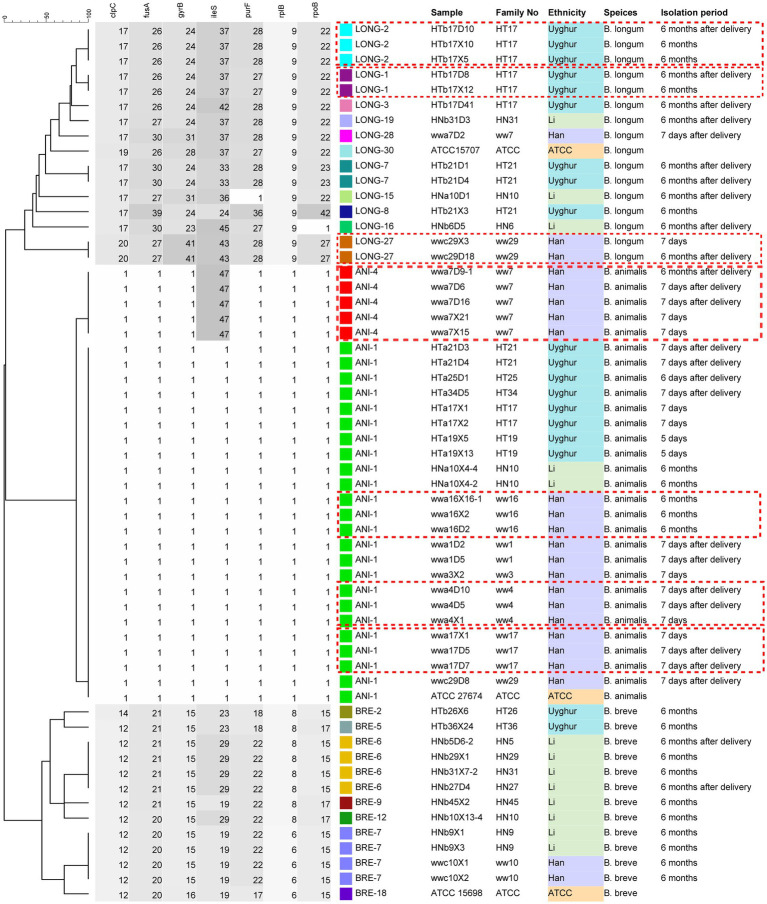
Dendrogram derived from a comparison of MLST profiles of *B. longum*, *B. breve*, and *B. animalis* isolates from feces of mothers and infants. The dendrogram was generated with a multiscale setting for comparison and UPGMA for clustering. The red boxes indicate strains isolated from feces of mothers and infants showing the same MLST profiles within a given cluster.

We further analyzed the MLST lineages of key bifidobacterial phylotypes frequently isolated from the three ethnic groups, including *B. longum* subsp. *longum*, *B. animalis* subsp*. lactis* and *B. breve*. By combining the 7 housekeeping genes, 20 distinct sequence types (*B. longum* subsp. *longum*, 11 STs; *B. breve*, 7 STs; *B.animalis* subsp*. lactis*, 2 STs) were identified in 55 strains. This result suggested a high genetic diversity in key bifidobacterial phylotypes. The results of the minimum spanning tree analysis revealed that specific STs were exclusively detected in particular ethnic groups (Figure 6B). Specifically, ST BRE-6, BRE-7, BRE-9, BRE-12, LONG-9, LONG-15, and LONG-16 were solely identified in the Li ethnic group; ST BRE-2, BRE-5, LONG-1, LONG-2, LONG-3, LONG-4, LONG-7 and LONG-8 were exclusively found in the Uyghur ethnic group; while, ST ANI-4, LONG-27, and LONG-28 were uniquely detected in the Han ethnic group. The result of the UPGMA dendrogram based on MLST profiles of 55 strains further showed that the mother and corresponding infant shared the same ST type. For example, strains wwa7D9–1, wwa7D6, wwa7D16, wwa7x21, and wwa7x15 isolated from the family ww7 shared the ST ANI -4 within a given cluster, and families ww4, ww16, and ww17 shared the ST ANI -1. Family HT17 shared two ST types of *Bifidobacterium longum*, including LONG-1 and LONG-2; wwc29X3 and wwc29D18, isolated from family ww29 shared the ST LONG-27 (Figure 7).

## Discussion

Bifidobacteria are well-recognized commensal bacteria in the human gut and are believed to interact with the host (Xiao et al., 2024; Turroni et al., 2021). It is also among the genera with the highest species diversity and intraspecific genetic diversity in the mammalian and human gut microbiota (Li W. et al. 2024; Deb, 2022; Pasolli et al., 2024; Duranti et al., 2016). However, *Bifidobacterium* is not the dominant bacterium in the adult gut, and some of its members belong to rare taxa that are difficult to detect even with metagenomic methods (Wang et al., 2021; Pasolli et al., 2019; Bogaert et al., 2023). To date, it is challenging to comprehensively and precisely elucidate the variability in bifidobacterial taxa profiles among human populations, particularly within mother-infant dyad cohorts.

### The *groEL* gene enables a more realistic reflection of the composition picture of the bifidobacterial community

Recently, several studies have reported that 16S full-length gene sequences can improve discrimination between bacterial species and distinguish strains or subspecies according to gene polymorphisms (Johnson et al., 2019). In our study, we first took advantage of the full-length sequence of the 16S rRNA (~1,500 bp) gene in combination with a third-generation sequencing platform (PacBio) to provide high-fidelity species and strain-level microbiota data as much as possible (He et al., 2020; Mosher et al., 2013; Callahan et al., 2019). However, as expected, using the standard universal forward primer 27f specific for bacteria resulted in remarkable underrepresentation of the sequences corresponding to bifidobacteria in the full-length rRNA gene data, as shown by other short sequences metadata (16S rRNA V4 and V4-V5 region), with only 0.57% taxa classified to species-level (Yan et al., 2021). The relative abundance of bifidobacteria taxa was only 1.92% in the infant group vs. 0.31% in the mother group (Figure 2D). In fact, it has been confirmed that more than 40% of purified Actinobacteria isolates failed to amplify with “universal” primers (Walker et al., 2015; Farris and Olson, 2007).

Based on very accurate and painstaking NCBI Blast analyses of the 16S rRNA gene dataset, our results showed that 26 unique ASVs were definitely assigned to 7 recognized *Bifidobacterium* species (Figure 2F). Unfortunately, our results were unable to mark *Bifidobacterium* at the subspecies level. Accumulated evidence has demonstrated that taxonomic identification at the strain or subspecies level based solely on the 16S rRNA gene is limited and insufficiently reliable (Enav et al., 2022; Li et al., 2025; Flores Ventura et al., 2025). By using bifidobacteria-specific primers such as the *groEL* and *tuf* genes, respectively, we sought to gain a more complete understanding of the *Bifidobacterium* community in the gut microbiota across and within mother-infant cohorts. In the *groEL* gene dataset, 743 ASVs obtained were categorized into 10 distinct *Bifidobacterium* species, along with 5 subspecies. Analogously, 827 ASVs belonging to 9 species and 8 subspecies were distinguished based on the *tuf* gene dataset (Figure 4). Both non-ribosomal genes were able to identify *Bifidobacterium* species at the subspecies level, especially the *tuf* gene, which can distinguish subspecies of the *B. longum* group. Nevertheless, performing an accurate Blast comparison revealed a flaw: reads of *B. longum* subsp. *longum* strain in the mock *Bifidobacterium* community was misclassified as subsp. *infantis* in the *tuf* dataset. This leads to the over-representation of the average relative abundance of *B. longum* subsp. *infantis* in the adult gut (14.24%), which accounts for merely 1.42% in the *groEL* dataset. Clearly, the latter is a more realistic reflection of the relative abundance of *B. longum* subsp. *infantis* in the adult gut. In fact, in the adult gut, *B. longum* subsp. *infantis*, a typical infant-type bifidobacterial phylotype, is rarely documented to date (Derrien et al., 2022; Barker-Tejeda et al., 2024). In our study, we isolated *B. longum* subsp. *infantis* strains from infant feces. Despite our best efforts, we were unable to recover *B. longum* subsp. *infantis* from the corresponding mother’s gut. Overall, our findings showed that sequencing of the *groEL* gene offers enhanced resolution for identifying bifidobacteria, thereby providing a more realistic representation of the compositional landscape of gut bifidobacterial microbiota.

### The core species composition of bifidobacteria in the gut shows distinct population-specific patterns across different ethnic groups

In general, human gut-associated *Bifidobacterium* species are recognized as human-residential bifidobacteria (HRB; Wong et al., 2020). Studies using culture methods have detected only a small number of bifidobacteria, with little variation in their composition across populations (Feehily et al., 2023; Wu et al., 2025). In fact, compared to metagenomic methods, functional gene amplicon sequencing can uncover a more comprehensive diversity pattern of the *Bifidobacterium* community, especially in adult microbiota. The concordant identification of key infant-type bifidobacteria (*B. longum* subsp. *infantis*, *B. bifidum,* and *B. breve*, etc.) by both the *groEL* and *tuf* markers not only validates the robustness of our profiling approach but also highlights their ecological dominance. Their near-universal presence (>90% prevalence) suggests that they are not mere passengers but likely keystone taxa foundational to early gut microbiota assembly and function (Li M. et al., 2024; Wu et al., 2025). Therefore, in terms of the occurrence and ecological distribution of HRB species, there should not be a very strict infant vs. adult subdivision. Based on an 80% prevalence, our findings support the notion that a core bifidobacterial taxa exists in the human gut across diverse populations, typically including *B. longum* subsp. *longum*, *B. longum* subsp. *infantis*, *B. pseudocatenulatum*, *B. breve*, *B. bifidum*, *B. adolescentis*, *B. catenulatum* subsp. *kashiwanohense*, *B. pseudolongum*, *B. animalis* subsp. *lactis*, *B. dentium*. In infant groups across the three cohorts, there was little difference in *Bifidobacterium* species profile in the two breastfeeding stages, but the proportions of different species varied substantially. Proficient HMO-using infant-type *Bifidobacterium* species, *B. longum*, *B. breve*, and *B. bifidum* were more abundant at 4–6 months of breastfeeding, especially *B. longum* subsp. *infantis* being significantly increased (Du et al., 2025). To our surprise, *B. animalis* is the dominant bifidobacterial taxon in the newborn gut during the initial stages of breastfeeding, which was inconsistent with results by other researchers, who reported that *B. longum* and *B. breve* had the highest relative abundance and prevalence within the first week of life (Saturio et al., 2021; An et al., 2024). One more plausible explanation is that human ancestors were chronically exposed to livestock and/or their habitats under agricultural conditions, leading to the expansion of the ecological niche of this bifidobacterial species (Ruebel et al., 2021; Tian et al., 2023).

In the current study, for strain-level analysis of the *Bifidobacterium* community, the amplicon library was processed with DADA2 to generate unique amplicon sequence variants (*groEL-*ASVs and *tuf*-ASVs), as in other studies (Yan et al., 2021; Mukherjee et al., 2021). The resulting dataset revealed high sequence-level diversity (microdiversity) based on the *groEL* gene in each *Bifidobacterium* species, such as *B. longum* ssp*. infantis* (44 ASVs) and *B. longum* ssp*. longum* (77 ASVs), *B. bifidum* (87 ASVs), *B. breve* (126 ASVs). These results support the hypothesis that the human-associated microbiota is often a complex mixture of closely related strains of the same species (Albanese and Donati, 2017; Heidrich et al., 2025). Of particular concern, we found cases in which more than two ASVs (strains) of the same species were present in some mother-infant pair or individual. Some studies suggest that the presence of several ASVs within a species favors its persistence across changing environmental conditions (García-García et al., 2019; Andreu-Sánchez et al., 2025). However, it remains unclear whether sequence-level variation within the same species corresponds to functionally distinct ecotypes that objectively exist. Moreover, the extent to which *Bifidobacterium* communities undergo dynamic shifts in strain composition in response to environmental/host changes is less well understood. Understanding these could guide microbiome-targeted therapies, including the selection or engineering of therapeutic bacteria for long-term colonization.

### Taxonomic profile of *Bifidobacterium* exhibiting population-specific characteristics linked to ethnic background in mother-infant transmission

Traditionally, species have been considered to be the basic unit for measuring microbial diversity (García-García et al., 2019). A growing body of evidence suggests that most bacterial species in human-associated microbial communities typically encompass strains with highly variable gene content (Van Rossum et al., 2020; Ellegaard et al., 2020). Strain-level diversity may contribute to discrepancies in certain species associations with health and disease, presenting cross-population microbial signatures in the form of a single strain or a combination of strains (Mei et al., 2024; Olm et al., 2022). Across a range of small cohort studies, it has been established that different infants infrequently harbor an identical bifidobacterial strain, even among twins (Zhang et al., 2015). The most conspicuous trend is that infants and their mothers exhibit a high degree of strain similarity, sharing the same strains in early and late lactation (at two time points during lactation), indicating persistence of strain transmission (Li W. et al., 2024; Li M. et al., 2024).

In the gut microbiota of great apes, certain lineages of *Bifidobacterium* were substantiated to co-evolve with their hosts (Nishida and Ochman, 2019). A more recent study showed that some gut microbial taxa have phylogenies that are more similar to the human phylogeny, providing evidence of codiversification to varying degrees (Suzuki et al., 2022). These findings highlight the significance of understanding the potential function of host individual- or population-specific microbial strains. In our study, using a culture-based approach, we found that the strain diversity of single *Bifidobacterium* species (the two most prevalent species, *B. longum* subsp. *longum* and *B. breve*) showed population-specificity across ethnic groups from different geographic regions (Figure 6B). Conspecific strains from the same mother–child pair are prone to being assigned to the same monophyletic group. Within a species, strains can show marked population specificity, shaped by shared ethnicity, living environment, dietary habits, and cultural practices. According to MAG-based phylogenies, four *Bifidobacterium* species showed evidence of codiversification in children (Suzuki et al., 2022). However, this is not the case for all symbiont bacteria in the gut microbiota. Our results indicated that almost all strains of *B. animalis* subsp. *lactis* belong to the same ST or genotype regardless of their ethnic source, which coincided with the observation of Milani et al., who reported that the *B. animalis* subsp. The *lactis* strain has a strictly monomorphic nature, showing no co-diversification with host sources (Milani et al., 2013). Similarly, some typical symbiont bacteria also showed the least evidence of cophylogeny with the human host.

Furthermore, our data showed that the same strains were present in the microbiota of mothers and unrelated individuals, supporting strain sharing within cohorts. Overall, based on the *groEL* and *tuf* genes metataxonomic datasets, the similarity of strain types within a sympatric mother-infant dyad cohort was significantly higher than that across mother-infant dyad cohorts, supporting the hypothesis of population specificity for the symbiont community. For mother-infant pairs, strain sharing is often touted as vertical transmission, a phenomenon that can be divided into in utero transmission (prenatal transfer) and postnatal mother-to-child transfer (postpartum transfer via breastfeeding or maternal contact; Browne et al., 2022; Xiao and Zhao, 2023; Meng et al., 2025).

Within a limited geographic area, long-term intergenerational vertical transmission from mother to child can lead to patterns of codiversification within a cohort. Despite the insufficient evidence for the horizontal transmission of *Bifidobacterium* species, strain transmission among related and unrelated individuals could potentially contribute to these patterns. Due to coexistence driven by dietary cross-feeding behavior, the strain community of various bifidobacterial species is likely to be tilted toward a highly host population-specific configuration (ethnicity), thereby forging characteristic metabolic pathways that are beneficial to indigenous populations, especially infants (Luo et al., 2021; Lou et al., 2023). GLM analysis revealed a negative correlation between maternal BMI and the relative abundance of *Bifidobacterium* (Supplementary Figure S3). Low *Bifidobacterium* levels are closely associated with obesity and other metabolic disorders, which is consistent with the negative trend observed in our study (Xiao et al., 2024). Maternal obesity has also been linked to the early acquisition of late-colonizing taxa in the neonatal gut microbiota (Corona-Cervantes et al., 2026).

Therefore, during the perinatal and lactation periods, it is feasible to modulate the maternal gut microbiota via diet and/or a combination of locally sourced probiotics (Sindi et al., 2021) to shape the establishment of a healthy gut microbiota in newborns and infants of the indigenous population more effectively (Manara et al., 2023). It should be noted that the present study lacked quantitative dietary data on maternal dietary intake during pregnancy and lactation. Future studies incorporating longitudinal dietary assessments are warranted to verify further the direct effects of diet on the maternal microbiota and the indirect regulatory role on the infant gut microbiome.

However, gene amplicon sequencing for investigating microbiota was still limited in its ability to resolve distinct microbial strains (Enav et al., 2022; Li et al., 2025). Although full-length 16S rRNA gene sequencing improves resolution compared with short-read approaches, it still does not enable definitive identification of biologically defined strains (Flores Ventura et al., 2025). To elucidate the currently unclear human population-specific and symbiont strain-specific mechanisms of host benefit, one issue of particular concern is whether the *Bifidobacterium* microbiota exhibits significant ecological specificity at the level of strain taxonomic resolution when species distributions are considered across multiple mother-infant dyad cohorts. Given that strain profiling can reliably assess functional implications, our findings highlight the need for genome-level validation to confirm strain identity and mother-to-infant transmission events. We next seek to perform deep metagenomic sequencing on a larger scale in human populations, aiming at a more detailed strain-level analysis of mother-infant dyad microbiota. Additionally, longitudinal tracking of mother-infant cohorts, whole-genome SNP analysis, and monitoring of potential shared environmental sources are crucial for rigorously distinguishing vertical transmission from environmental exposure and clarifying factors that shape microbial community assembly.

## Data Availability

The datasets presented in this study can be found in online repositories. The names of the repository/repositories and accession number(s) can be found in the article/Supplementary material.

## Footnotes

1
http://www.cpndb.ca

2
http://www.ehbio.com/test/venn

## References

[ref1] Afzaal M. Saeed F. Shah Y. A. Hussain M. Rabail R. Socol C. T. . (2022). Human gut microbiota in health and disease: unveiling the relationship. Front. Microbiol. 13:999001. doi: 10.3389/fmicb.2022.999001, 36225386 PMC9549250

[ref2] Albanese D. Donati C. (2017). Strain profiling and epidemiology of bacterial species from metagenomic sequencing. Nat. Commun. 8:2260. doi: 10.1038/s41467-017-02209-5, 29273717 PMC5741664

[ref3] Alcon-Giner C. Caim S. Mitra S. Ketskemety J. Wegmann U. Wain J. . (2017). Optimisation of 16s rrna gut microbiota profiling of extremely low birth weight infants. BMC Genomics 18:841. doi: 10.1186/s12864-017-4229-x, 29096601 PMC5668952

[ref4] Alessandri G Ossiprandi MC Ventura M van Sinderen D (2021). Protocol to Select Bifidobacteria From Fecal and Environmental Samples. Methods Mol. Biol. (Clifton, N.J.), 2278, 61–70. doi: 10.1007/978-1-0716-1274-3_6 33649948

[ref5] An R. Fontana F. Van Daele E. Ventura M. Vlieger A. van Elburg R. M. . (2024). Longitudinal changes in bifidobacterial population during the first two years of life. Benefic. Microbes 15, 227–240. doi: 10.1163/18762891-bja00012, 38677714

[ref6] Andreu-Sánchez S. Blanco-Míguez A. Wang D. Golzato D. Manghi P. Heidrich V. . (2025). Global genetic diversity of human gut microbiome species is related to geographic location and host health. Cell 188, 3942–3959.e9. doi: 10.1016/j.cell.2025.04.014, 40311618

[ref7] Barker-Tejeda T. C. Zubeldia-Varela E. Macías-Camero A. Alonso L. Martín-Antoniano I. A. Rey-Stolle M. F. . (2024). Comparative characterization of the infant gut microbiome and their maternal lineage by a multi-omics approach. Nat. Commun. 15:3004. doi: 10.1038/s41467-024-47182-y, 38589361 PMC11001937

[ref8] Bogaert D. van Beveren G. J. de Koff E. M. Lusarreta Parga P. Balcazar Lopez C. E. Koppensteiner L. . (2023). Mother-to-infant microbiota transmission and infant microbiota development across multiple body sites. Cell Host Microbe 31, 447–460.e6. doi: 10.1016/j.chom.2023.01.018, 36893737

[ref9] Browne H. P. Shao Y. Lawley T. D. (2022). Mother–infant transmission of human microbiota. Curr. Opin. Microbiol. 69:102173. doi: 10.1016/j.mib.2022.102173, 35785616

[ref10] Callahan B. J. Wong J. Heiner C. Oh S. Theriot C. M. Gulati A. S. . (2019). High-throughput amplicon sequencing of the full-length 16S rRNA gene with single-nucleotide resolution. Nucleic Acids Res. 47:e103. doi: 10.1093/nar/gkz569, 31269198 PMC6765137

[ref11] Casaburi G. Duar R. M. Brown H. Mitchell R. D. Kazi S. Chew S. . (2021). Metagenomic insights of the infant microbiome community structure and function across multiple sites in the United States. Sci. Rep. 11:1472. doi: 10.1038/s41598-020-80583-9, 33479326 PMC7820601

[ref12] Chen K. Wang H. Yang Y. Tang C. Sun X. Zhou J. . (2025). Common mechanisms of gut microbe-based strategies for the treatment of intestine-related diseases: based on multi-target interactions with the intestinal barrier. Cell Commun. Signal 23:288. doi: 10.1186/s12964-025-02299-5, 40528179 PMC12175372

[ref13] Cheng M. Ge X. Zhong C. Fu R. Ning K. Xu S. (2021). Micro-coevolution of host genetics with gut microbiome in three chinese ethnic groups. J. Genet. Genomics 48, 972–983. doi: 10.1016/j.jgg.2021.09.002, 34562635

[ref14] Corona-Cervantes K. Urrutia-Baca V. H. Gámez-Valdez J. S. Jiménez-López B. Rodríguez-Gutierrez N. A. Chávez-Caraza K. . (2026). Maternal obesity alters human milk oligosaccharides content and correlates with early acquisition of late colonizers in the neonatal gut microbiome. Gut Microbes 18:2607043. doi: 10.1080/19490976.2025.2607043 41536238 PMC12818807

[ref15] Culp E. J. Goodman A. L. (2023). Cross-feeding in the gut microbiome: ecology and mechanisms. Cell Host Microbe 31, 485–499. doi: 10.1016/j.chom.2023.03.016, 37054671 PMC10125260

[ref16] Deb S. (2022). Pan-genome evolution and its association with divergence of metabolic functions in bifidobacterium genus. World J. Microbiol. Biotechnol. 38:231. doi: 10.1007/s11274-022-03430-1, 36205822

[ref17] Dehingia M. Adak A. Khan M. R. (2019). Ethnicity-influenced microbiota: a future healthcare perspective. Trends Microbiol. 27, 191–193. doi: 10.1016/j.tim.2019.01.002, 30685243

[ref18] Delétoile A. Passet V. Aires J. Chambaud I. Butel M.-J. Smokvina T. . (2010). Species delineation and clonal diversity in four bifidobacterium species as revealed by multilocus sequencing. Res. Microbiol. 161, 82–90. doi: 10.1016/j.resmic.2009.12.006, 20060895

[ref19] Derrien M. Turroni F. Ventura M. van Sinderen D. (2022). Insights into endogenous bifidobacterium species in the human gut microbiota during adulthood. Trends Microbiol. 30, 940–947. doi: 10.1016/j.tim.2022.04.004, 35577716

[ref20] Du Y. Cheng J. Xie R. Zhang Y. Huang Z. Jin G. . (2025). The triad of maternal gut-breast milk-infant gut microbial transmission in early life as a critical pathway for microbial inheritance. Gut Microbes 17:2574928. doi: 10.1080/19490976.2025.2574928, 41243311 PMC12629333

[ref21] Duranti S. Milani C. Lugli G. A. Mancabelli L. Turroni F. Ferrario C. . (2016). Evaluation of genetic diversity among strains of the human gut commensal bifidobacterium adolescentis. Sci. Rep. 6:23971. doi: 10.1038/srep23971, 27035119 PMC4817515

[ref22] Ellegaard K. M. Suenami S. Miyazaki R. Engel P. (2020). Vast differences in strain-level diversity in the gut microbiota of two closely related honey bee species. Curr. Biol. 30, 2520–2531.e7. doi: 10.1016/j.cub.2020.04.070, 32531278 PMC7342003

[ref23] Enav H. Bäckhed F. Ley R. E. (2022). The developing infant gut microbiome: a strain-level view. Cell Host Microbe 30, 627–638. doi: 10.1016/j.chom.2022.04.009, 35550666

[ref24] Farris M. H. Olson J. B. (2007). Detection of actinobacteria cultivated from environmental samples reveals bias in universal primers. Lett. Appl. Microbiol. 45, 376–381. doi: 10.1111/j.1472-765X.2007.02198.x, 17897379

[ref25] Feehily C. O’Neill I. J. Walsh C. J. Moore R. L. Killeen S. L. Geraghty A. A. . (2023). Detailed mapping of bifidobacterium strain transmission from mother to infant via a dual culture-based and metagenomic approach. Nat. Commun. 14:3015. doi: 10.1038/s41467-023-38694-0, 37230981 PMC10213049

[ref26] Flores Ventura E. Esteban-Torres M. Gueimonde M. van Sinderen D. Koren O. Hall L. J. . (2025). Mother-to-infant vertical transmission in early life: a systematic review and proportional meta-analysis of bifidobacterium strain transmissibility. NPJ Biofilms Microbiomes 11:121. doi: 10.1038/s41522-025-00720-y, 40593735 PMC12219069

[ref27] García-García N. Tamames J. Linz A. M. Pedrós-Alió C. Puente-Sánchez F. (2019). Microdiversity ensures the maintenance of functional microbial communities under changing environmental conditions. ISME J. 13, 2969–2983. doi: 10.1038/s41396-019-0487-8, 31417155 PMC6864100

[ref28] Groussin M. Mazel F. Alm E. J. (2020). Co-evolution and co-speciation of host-gut bacteria systems. Cell Host Microbe 28, 12–22. doi: 10.1016/j.chom.2020.06.013, 32645351

[ref29] He Q. Kwok L.-Y. Xi X. Zhong Z. Ma T. Xu H. . (2020). The meconium microbiota shares more features with the amniotic fluid microbiota than the maternal fecal and vaginal microbiota. Gut Microbes 12:1794266. doi: 10.1080/19490976.2020.1794266, 32744162 PMC7524391

[ref30] Heidrich V. Valles-Colomer M. Segata N. (2025). Human microbiome acquisition and transmission. Nat. Rev. Microbiol. 23, 568–584. doi: 10.1038/s41579-025-01166-x, 40119155

[ref31] Hoang D. T. Chernomor O. Von Haeseler A. Minh B. Q. Vinh L. S. (2018). UFBoot2: improving the ultrafast bootstrap approximation. Mol. Biol. Evol. 35, 518–522. doi: 10.1093/molbev/msx281, 29077904 PMC5850222

[ref32] Hu L. Lu W. Wang L. Pan M. Zhang H. Zhao J. . (2017). Assessment of bifidobacterium species using groel gene on the basis of illumina miseq high-throughput sequencing. Gene 8:336. doi: 10.3390/genes8110336, 29160815 PMC5704249

[ref33] Johnson J. S. Spakowicz D. J. Hong B.-Y. Petersen L. M. Demkowicz P. Chen L. . (2019). Evaluation of 16s rRNA gene sequencing for species and strain-level microbiome analysis. Nat. Commun. 10:5029. doi: 10.1038/s41467-019-13036-1, 31695033 PMC6834636

[ref34] Kurilshikov A. Medina-Gomez C. Bacigalupe R. Radjabzadeh D. Wang J. Demirkan A. . (2021). Large-scale association analyses identify host factors influencing human gut microbiome composition. Nat. Genet. 53, 156–165. doi: 10.1038/s41588-020-00763-1, 33462485 PMC8515199

[ref35] Lanfear R. Von Haeseler A. Woodhams M. D. Schrempf D. Chernomor O. Schmidt H. A. . (2020). IQ-TREE 2: new models and efficient methods for phylogenetic inference in the genomic era. Mol. Biol. Evol. 37, 1530–1534. doi: 10.1093/molbev/msaa015, 32011700 PMC7182206

[ref36] Laursen M. F. Roager H. M. (2023). Human milk oligosaccharides modify the strength of priority effects in the bifidobacterium community assembly during infancy. ISME J. 17, 2452–2457. doi: 10.1038/s41396-023-01525-7, 37816852 PMC10689826

[ref37] Letunic I. Bork P. (2016). Interactive tree of life (itol) v3: An online tool for the display and annotation of phylogenetic and other trees. Nucleic Acids Res. 44, W242–W245. doi: 10.1093/nar/gkw290, 27095192 PMC4987883

[ref38] Li W. Liang H. He W. Gao X. Wu Z. Hu T. . (2024). Genomic and functional diversity of cultivated bifidobacterium from human gut microbiota. Heliyon 10:e27270. doi: 10.1016/j.heliyon.2024.e27270, 38463766 PMC10923715

[ref39] Li Y. Shi Z. Zhang X. Ren H. Ji H. Yang F. . (2025). Metagenomic analysis revealing links between age, gut microbiota and bone loss in chinese adults. npj Metabolic Health Dis. 3:18. doi: 10.1038/s44324-025-00060-7, 40604305 PMC12118732

[ref40] Li M. Xue Y. Lu H. Bai J. Cui L. Ning Y. . (2024). Relationship between infant gastrointestinal microorganisms and maternal microbiome within 6 months of delivery. Spectrum 12:e0360823. doi: 10.1128/spectrum.03608-23, 39172626 PMC11448430

[ref41] Lin Y. W. Liu M. C. Lu T. J. Ho Y. Y. Chen T. W. Yang Y. J. (2025). The developmental trajectory and correlation of human Milk microbiota and oligosaccharides in Taiwanese lactating mothers. J. Food Sci. 90:e70484. doi: 10.1111/1750-3841.70484, 40814727

[ref42] Liu S. Liu Q. Pan C. Morrin S. T. Buck R. H. Li X. . (2026). New insights into human milk oligosaccharide profiles in China: findings from a large-scale analysis of human milk. Nutrients 18:417. doi: 10.3390/nu18030417, 41683240 PMC12899140

[ref43] Liu Y.-X. Qin Y. Chen T. Lu M. Qian X. Guo X. . (2020). A practical guide to amplicon and metagenomic analysis of microbiome data. Protein Cell 12, 315–330. doi: 10.1007/s13238-020-00724-8, 32394199 PMC8106563

[ref44] Lou Y. C. Rubin B. E. Schoelmerich M. C. DiMarco K. S. Borges A. L. Rovinsky R. . (2023). Infant microbiome cultivation and metagenomic analysis reveal bifidobacterium 2′-fucosyllactose utilization can be facilitated by coexisting species. Nat. Commun. 14:7417. doi: 10.1038/s41467-023-43279-y, 37973815 PMC10654741

[ref45] Lu J. Zhang L. Zhai Q. Zhao J. Zhang H. Lee Y.-K. . (2021). Chinese gut microbiota and its associations with staple food type, ethnicity, and urbanization. NPJ Biofilms Microbiomes 7:71. doi: 10.1038/s41522-021-00245-0, 34489454 PMC8421333

[ref46] Lugli G. A. Alessandri G. Milani C. Mancabelli L. Ruiz L. Fontana F. . (2020). Evolutionary development and co-phylogeny of primate-associated bifidobacteria. Environ. Microbiol. 22, 3375–3393. doi: 10.1111/1462-2920.15108, 32515117

[ref47] Luo Y. Xiao Y. Zhao J. Zhang H. Chen W. Zhai Q. (2021). The role of mucin and oligosaccharides via cross-feeding activities by bifidobacterium: a review. Int. J. Biol. Macromol. 167, 1329–1337. doi: 10.1016/j.ijbiomac.2020.11.087, 33202267

[ref48] Maher S. E. O’Brien E. C. Moore R. L. Byrne D. F. Geraghty A. A. Saldova R. . (2020). The association between the maternal diet and the maternal and infant gut microbiome: a systematic review. Br. J. Nutr. 129, 1491–1499. doi: 10.1017/s0007114520000847, 32129734

[ref49] Makino H. Kushiro A. Ishikawa E. Muylaert D. Kubota H. Sakai T. . (2011). Transmission of intestinal *bifidobacterium longum* subsp. Longum strains from mother to infant, determined by multilocus sequencing typing and amplified fragment length polymorphism. Appl. Environ. Microbiol. 77, 6788–6793. doi: 10.1128/aem.05346-11, 21821739 PMC3187114

[ref50] Manara S. Selma-Royo M. Huang K. D. Asnicar F. Armanini F. Blanco-Miguez A. . (2023). Maternal and food microbial sources shape the infant microbiome of a rural ethiopian population. Curr. Biol. 33, 1939–1950.e4. doi: 10.1016/j.cub.2023.04.011, 37116481 PMC10234599

[ref51] Mei Z. Wang F. Bhosle A. Dong D. Mehta R. Ghazi A. . (2024). Strain-specific gut microbial signatures in type 2 diabetes identified in a cross-cohort analysis of 8,117 metagenomes. Nat. Med. 30, 2265–2276. doi: 10.1038/s41591-024-03067-7, 38918632 PMC11620793

[ref52] Meng L. Xie H. Li Z. Tye K. D. Fan G. Huang T. . (2025). Gut-mammary pathway: breast milk microbiota as a mediator of maternal gut microbiota transfer to the infant gut. J. Funct. Foods 124:106620. doi: 10.1016/j.jff.2024.106620

[ref53] Milani C. Duranti S. Lugli G. A. Bottacini F. Strati F. Arioli S. . (2013). Comparative genomics of bifidobacterium animalis subsp. Lactis reveals a strict monophyletic bifidobacterial taxon. Appl. Environ. Microbiol. 79, 4304–4315. doi: 10.1128/aem.00984-13, 23645200 PMC3697524

[ref54] Moeller A. H. S. T. Phifer-Rixey M. Nachman M. W. (2018). Transmission modes of the mammalian gut microbiota. Science 362, 453–457. doi: 10.1126/science.aat7164, 30361372

[ref55] Mosher J. J. Bernberg E. L. Shevchenko O. Kan J. Kaplan L. A. (2013). Efficacy of a 3rd generation high-throughput sequencing platform for analyses of 16S rRNA genes from environmental samples. J. Microbiol. Methods 95, 175–181. doi: 10.1016/j.mimet.2013.08.009, 23999276

[ref56] Mujagic Z. Kasapi M. Jonkers D. M. A. E. Garcia-Perez I. Vork L. Weerts Z. Z. R. M. . (2022). Integrated fecal microbiome–metabolome signatures reflect stress and serotonin metabolism in irritable bowel syndrome. Gut Microbes 14:2063016. doi: 10.1080/19490976.2022.2063016, 35446234 PMC9037519

[ref57] Mukherjee C. Moyer C. O. Steinkamp H. M. Hashmi S. B. Beall C. J. Guo X. . (2021). Acquisition of oral microbiota is driven by environment, not host genetics. Microbiome. 9:54. doi: 10.1186/s40168-020-00986-8, 33622378 PMC7903647

[ref58] Nishida A. H. Ochman H. (2019). A great-ape view of the gut microbiome. Nat. Rev. Genet. 20, 195–206. doi: 10.1038/s41576-018-0085-z, 30622302

[ref59] Olm M. R. Dahan D. Carter M. M. Merrill B. D. Yu F. B. Jain S. . (2022). Robust variation in infant gut microbiome assembly across a spectrum of lifestyles. Science 376, 1220–1223. doi: 10.1126/science.abj2972, 35679413 PMC9894631

[ref60] Pasolli E. Asnicar F. Manara S. Zolfo M. Karcher N. Armanini F. . (2019). Extensive unexplored human microbiome diversity revealed by over 150,000 genomes from metagenomes spanning age, geography, and lifestyle. Cell 176, 649–662.e20. doi: 10.1016/j.cell.2019.01.001, 30661755 PMC6349461

[ref61] Pasolli E. Mauriello I. E. Avagliano M. Cavaliere S. De Filippis F. Ercolini D. (2024). Bifidobacteriaceae diversity in the human microbiome from a large-scale genome-wide analysis. Cell Rep. 43:115027. doi: 10.1016/j.celrep.2024.115027, 39602306

[ref62] Qi C. Tu H. Zhou J. Tu R. Chang H. Chen J. . (2022). Widespread vertical transmission of secretory immunoglobulin a coated trace bacterial variants from the mother to infant gut through breastfeeding. Food Funct. 13, 11543–11554. doi: 10.1039/d2fo01244h, 36260082

[ref63] Reese A. T. Phillips S. R. Owens L. A. Venable E. M. Langergraber K. E. Machanda Z. P. . (2021). Age patterning in wild chimpanzee gut microbiota diversity reveals differences from humans in early life. Curr. Biol. 31, 613–620.e3. doi: 10.1016/j.cub.2020.10.075, 33232664 PMC7993011

[ref64] Rook O. Zwart H. (2025). Awareness of human microbiome may promote healthier lifestyle and more positive environmental attitudes. Commun. Med. 5:39. doi: 10.1038/s43856-025-00747-4, 39930028 PMC11811053

[ref65] Ruebel M. L. Gilley S. P. Sims C. R. Zhong Y. Turner D. Chintapalli S. V. . (2021). Associations between maternal diet, body composition and gut microbial ecology in pregnancy. Nutrients 13:3295. doi: 10.3390/nu13093295, 34579172 PMC8468685

[ref66] Saturio S. Nogacka A. M. Suárez M. Fernández N. Mantecón L. Mancabelli L. . (2021). Early-life development of the bifidobacterial community in the infant gut. Int. J. Mol. Sci. 22:3382. doi: 10.3390/ijms22073382, 33806135 PMC8036440

[ref67] Sindi A. S. Geddes D. T. Wlodek M. E. Muhlhausler B. S. Payne M. S. Stinson L. F. (2021). Can we modulate the breastfed infant gut microbiota through maternal diet? FEMS Microbiol. Rev. 45:fuab011. doi: 10.1093/femsre/fuab011, 33571360

[ref68] Singh R. P. (2019). Glycan utilisation system in bacteroides and bifidobacteria and their roles in gut stability and health. Appl. Microbiol. Biotechnol. 103, 7287–7315. doi: 10.1007/s00253-019-10012-z, 31332487

[ref69] Sun W. Zhang Y. Guo R. Sha S. Chen C. Ullah H. . (2024). A population-scale analysis of 36 gut microbiome studies reveals universal species signatures for common diseases. NPJ Biofilms Microbiomes 10:96. doi: 10.1038/s41522-024-00567-9, 39349486 PMC11442664

[ref70] Suzuki T. A. Fitzstevens J. L. Schmidt V. T. Enav H. Huus K. E. Mbong Ngwese M. . (2022). Codiversification of gut microbiota with humans. Science 377, 1328–1332. doi: 10.1126/science.abm7759, 36108023 PMC10777373

[ref72] Tian M. Li Q. Zheng T. Yang S. Chen F. Guan W. . (2023). Maternal microbe-specific modulation of the offspring microbiome and development during pregnancy and lactation. Gut Microbes 15:2206505. doi: 10.1080/19490976.2023.2206505, 37184203 PMC10187089

[ref73] Turroni F. van Sinderen D. Ventura M. (2021). Bifidobacteria: insights into the biology of a key microbial group of early life gut microbiota. Microbiome Res. Reports. 1:2. doi: 10.20517/mrr.2021.02, 38045555 PMC10688781

[ref74] Underwood M. A. Mukhopadhyay S. Lakshminrusimha S. Bevins C. L. (2020). Neonatal intestinal dysbiosis. J. Perinatol. 40, 1597–1608. doi: 10.1038/s41372-020-00829-2, 32968220 PMC7509828

[ref75] Van Rossum T. Ferretti P. Maistrenko O. M. Bork P. (2020). Diversity within species: interpreting strains in microbiomes. Nat. Rev. Microbiol. 18, 491–506. doi: 10.1038/s41579-020-0368-1, 32499497 PMC7610499

[ref76] Vlková E. Salmonová H. Bunešová V. Geigerová M. Rada V. Musilová Š. (2015). A new medium containing mupirocin, acetic acid, and norfloxacin for the selective cultivation of bifidobacteria. Anaerobe 34, 27–33. doi: 10.1016/j.anaerobe.2015.04.001, 25865525

[ref77] Walker A. W. Martin J. C. Scott P. Parkhill J. Flint H. J. Scott K. P. (2015). 16s rrna gene-based profiling of the human infant gut microbiota is strongly influenced by sample processing and pcr primer choice. Microbiome. 3:26. doi: 10.1186/s40168-015-0087-4, 26120470 PMC4482049

[ref78] Wang S. Zeng S. Egan M. Cherry P. Strain C. Morais E. . (2021). Metagenomic analysis of mother-infant gut microbiome reveals global distinct and shared microbial signatures. Gut Microbes 13, 1–24. doi: 10.1080/19490976.2021.1911571, 33960282 PMC8115609

[ref79] Wong C. B. Odamaki T. Xiao J.-z. (2020). Insights into the reason of human-residential bifidobacteria (hrb) being the natural inhabitants of the human gut and their potential health-promoting benefits. FEMS Microbiol. Rev. 44, 369–385. doi: 10.1093/femsre/fuaa010, 32319522 PMC7326374

[ref80] Wu S. Luo G. Jiang F. Jia W. Li J. Huang T. . (2025). Early life bifidobacterial mother–infant transmission: greater contribution from the infant gut to human milk revealed by microbiomic and culture-based methods. mSystems. 10:e0048025. doi: 10.1128/msystems.00480-25, 40558046 PMC12282193

[ref81] Xiao M. Zhang C. Duan H. Narbad A. Zhao J. Chen W. . (2024). Cross-feeding of bifidobacteria promotes intestinal homeostasis: a lifelong perspective on the host health. NPJ Biofilms Microbiomes 10:47. doi: 10.1038/s41522-024-00524-6, 38898089 PMC11186840

[ref82] Xiao L. Zhao F. (2023). Microbial transmission, colonisation and succession: from pregnancy to infancy. Gut 72, 772–786. doi: 10.1136/gutjnl-2022-328970, 36720630 PMC10086306

[ref83] Yan W. Luo B. Zhang X. Ni Y. Tian F. (2021). Association and occurrence of bifidobacterial phylotypes between breast milk and fecal microbiomes in mother–infant dyads during the first 2 years of life. Front. Microbiol. 12:669442. doi: 10.3389/fmicb.2021.669442, 34163448 PMC8215152

[ref84] Yang M. Chen T. Liu Y. X. Huang L. (2024). Visualizing set relationships: Evenn's comprehensive approach to venn diagrams. iMeta 3:e184. doi: 10.1002/imt2.184, 38898979 PMC11183158

[ref85] Yang B. Ding M. Chen Y. Han F. Yang C. Zhao J. . (2021). Development of gut microbiota and bifidobacterial communities of neonates in the first 6 weeks and their inheritance from mother. Gut Microbes 13, 1–13. doi: 10.1080/19490976.2021.1908100, 33847206 PMC8049200

[ref86] Yanokura E Oki K Makino H Modesto M Pot B Mattarelli P . (2015). Subspeciation of bifidobacterium longum by multilocus approaches and amplified fragment length polymorphism. Description of B. longum subsp. suillum subsp. nov., isolated from the faeces of piglets. Syst. Appl. Microbiol. 38, 305–314. doi: 10.1016/j.syapm.2015.05.001 26007614

[ref87] Zeng T. Zuo L. Yu Q. Wu Q. Bao Z. Xiong H. . (2026). Role and mechanisms of gut microbiota in infectious diseases: recent evidence from animal models. Biology 15: 256. doi: 10.3390/biology15030256, 41677726 PMC12897338

[ref88] Zhang M. Hang X. Tan J. Yang H. Schloss P. D. (2015). The host genotype and environment affect strain types of bifidobacterium longum subsp. Longum inhabiting the intestinal tracts of twins. Appl. Environ. Microbiol. 81, 4774–4781. doi: 10.1128/aem.00249-15, 25956768 PMC4551205

[ref89] Zhang B. Li L. Q. Liu F. Wu J. Y. (2022). Human milk oligosaccharides and infant gut microbiota: molecular structures, utilization strategies and immune function. Carbohydr. Polym. 276:118738. doi: 10.1016/j.carbpol.2021.118738, 34823774

[ref90] Zhao Z. Zhao F. Chimeddorj B. Sun Z. Tserenkhuu E. Ochirdanzan M. . (2025). Dietary nutrition, gut microbiota, and health status across geographically diverse populations in mongolia: a cross-sectional study. Food Sci. Nutr. 13:e70531. doi: 10.1002/fsn3.70531, 40621189 PMC12227796

[ref91] Zilber-Rosenberg I. Rosenberg E. (2021). Microbial-driven genetic variation in holobionts. FEMS Microbiol. Rev. 45:fuab022. doi: 10.1093/femsre/fuab022, 33930136

