# Supplementary material for: Significant strain microdiversity in mother-infant dyad cohorts across ethnic groups reveals population specificity of bifidobacteria microbiota transmission
Source: Front Microbiol. 2026 May 29;17:1814222. doi: 10.3389/fmicb.2026.1814222 (PMC13260250; doi:10.3389/fmicb.2026.1814222)
Supplement: Supplementary file 2 [file Table_2.DOCX]

**Significant strain microdiversity in mother-infant dyad cohorts across ethnic groups reveals population specificity of Bifidobacteria microbiota transmission**

Huimin Zhang^1^, Quanhao Zhao^1^, Baolong Luo^1^, Xueling Zhang^1^, Jian Huang^1^, Yanzhuan Lu^1^, Fengwei Tian^2^, Hailong Sun ^1^*, Yongqing Ni^1^*

1 School of Food Science and Technology/Key Laboratory for Xinjiang Special Probiotics and Dairy Technology of The Eighth Division of XPCC, Shihezi University, Shihezi 832003, China.

2 School of Food Science and Technology, Jiangnan University, Wuxi 214122, China.

*Correspondence: Yongqing Ni: [niyqlzu@sina.com](mailto:niyqlzu@sina.com), ORCID: 0000-0003-4876-589X, telephone: 15299950600 (Y.N.); Hailong Sun: [sunhl610@shzu.edu.cn](mailto:sunhl610@shzu.edu.cn), telephone: 13101978585 (H.S.).

**Supplementary materials**

**Figure S1** TM scores and the dissimilarity index between mother-infant dyads based on 16S rRNA gene. (A) Density plots of TM values for *Bacteroides*, *Bifidobacterium*, *Staphylococcus*, and *Streptococcus*, where dashed lines denote a TM score of 1. (B) Pairwise analysis of the dissimilarity between different groups based on the bray-curtis distance between related mother-infant dyads in the Han, Li and Uyghur ethnic groups at phylum, genus and species level. Every dot represents a pair, and the gray line represents samples from the same mother-infant dyad. Paired Wilcoxon-ranked sum test, Bonferroni corrected. FDR is controlled by the Benjamini-Hochberg method. *FDR <0.05, **FDR< 0.01, *** FDR < 0.005.

**
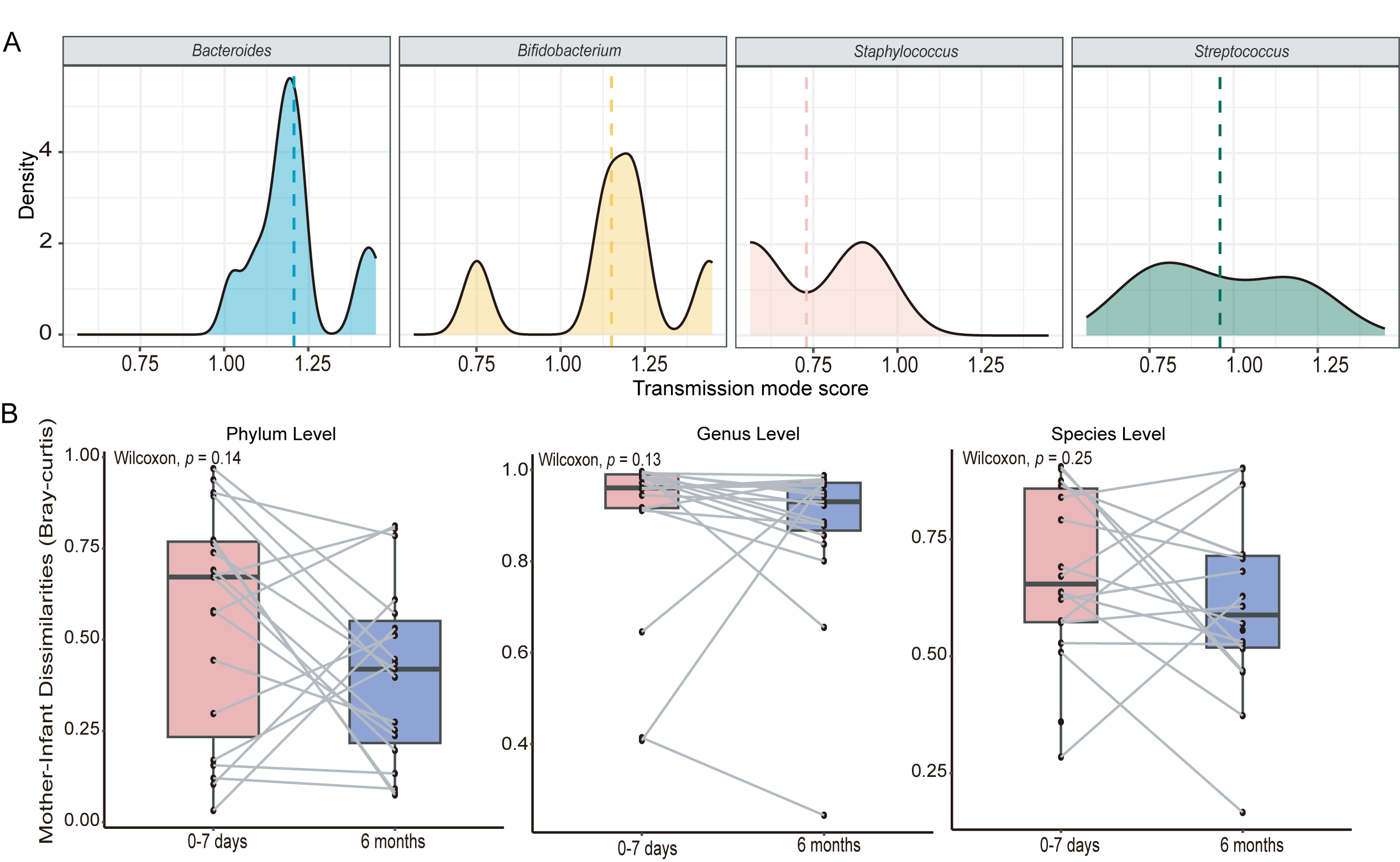
**

**Figure S2** Diversity and composition spectrum of *Bifidobacterium* across 54 mother-infant dyads from three distinct ethnic groups based on *tuf* gene. (A) The box plot of observed features and shannon index at species level. Nonmetric multidimensional scaling (NMDS) ordination based on bray-curtis distance of each group colored by lineage. Ellipses denote 90% confidence intervals. (B) Ternary map of species composition of *Bifidobacterium* based on *tuf* gene. The four figures are the neonatal group, the 6-month-old group, the group of mothers after delivery, and the 6-month postpartum mothers. (C) The dynamic changes of *B. aolescentis*, *B. pseudocatenulatum*, *B. animalis* group, *B. longum* group, *B. breve*, *and B. bifidum* detected in maternal and infant of different ethnic groups at 0-7 days and 6 months were analyzed, respectively. The figure shows the ratio of differences in the relative abundance of bifidobacterial species over 95% confidence intervals. (D) Heatmap of shared *Bifidobacterium* species of mother-infant pairs based on *tuf* gene.


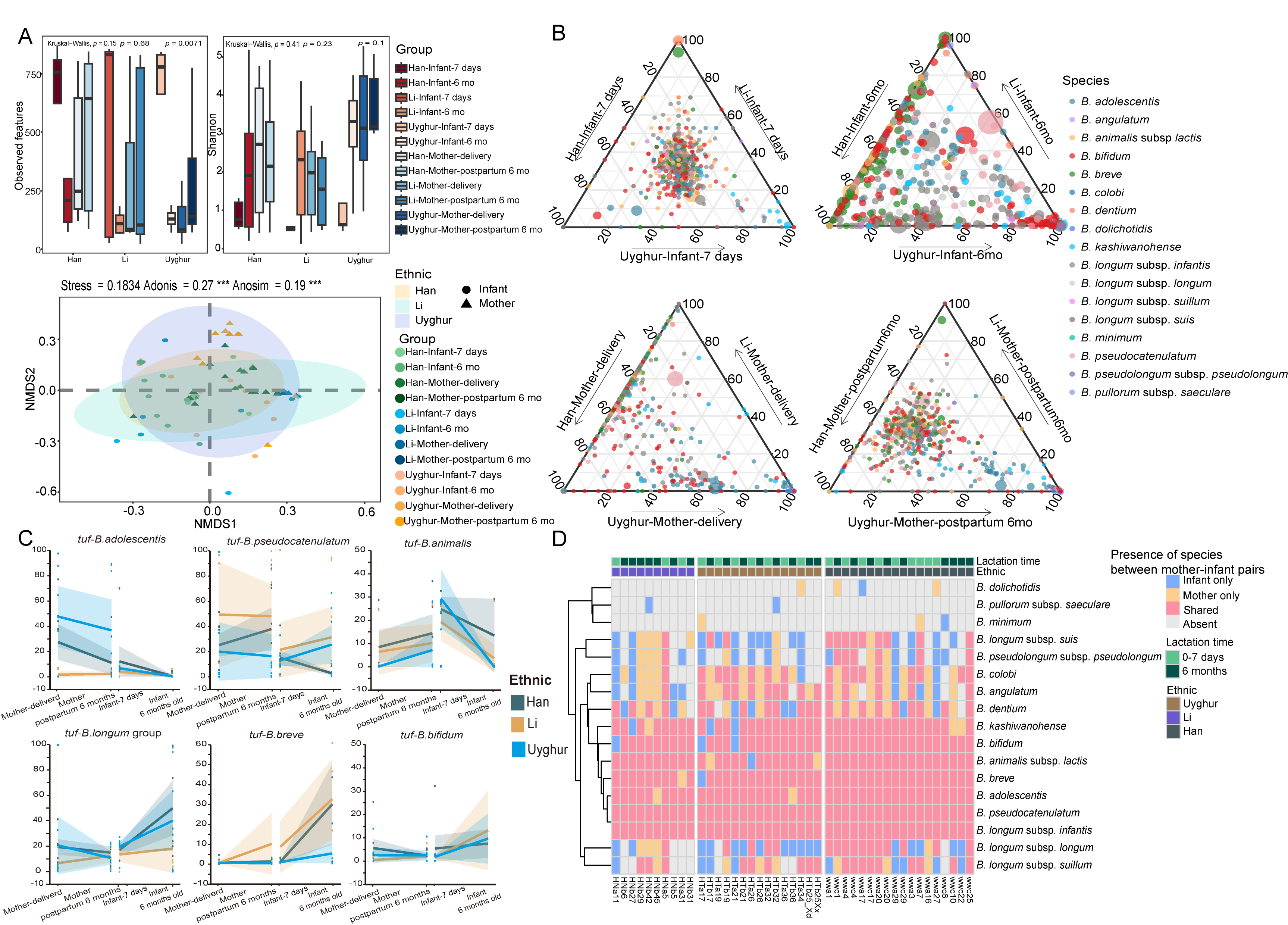


**Figure S3** Generalized linear model analysis of maternal BMI and the relative abundance of *Bifidobacterium*. R indicates the spearman correlation coefficient, and *p* represents the statistical significance. *Bifidobacterium* relative abundance was determined based on *groEL* gene amplicon sequencing.


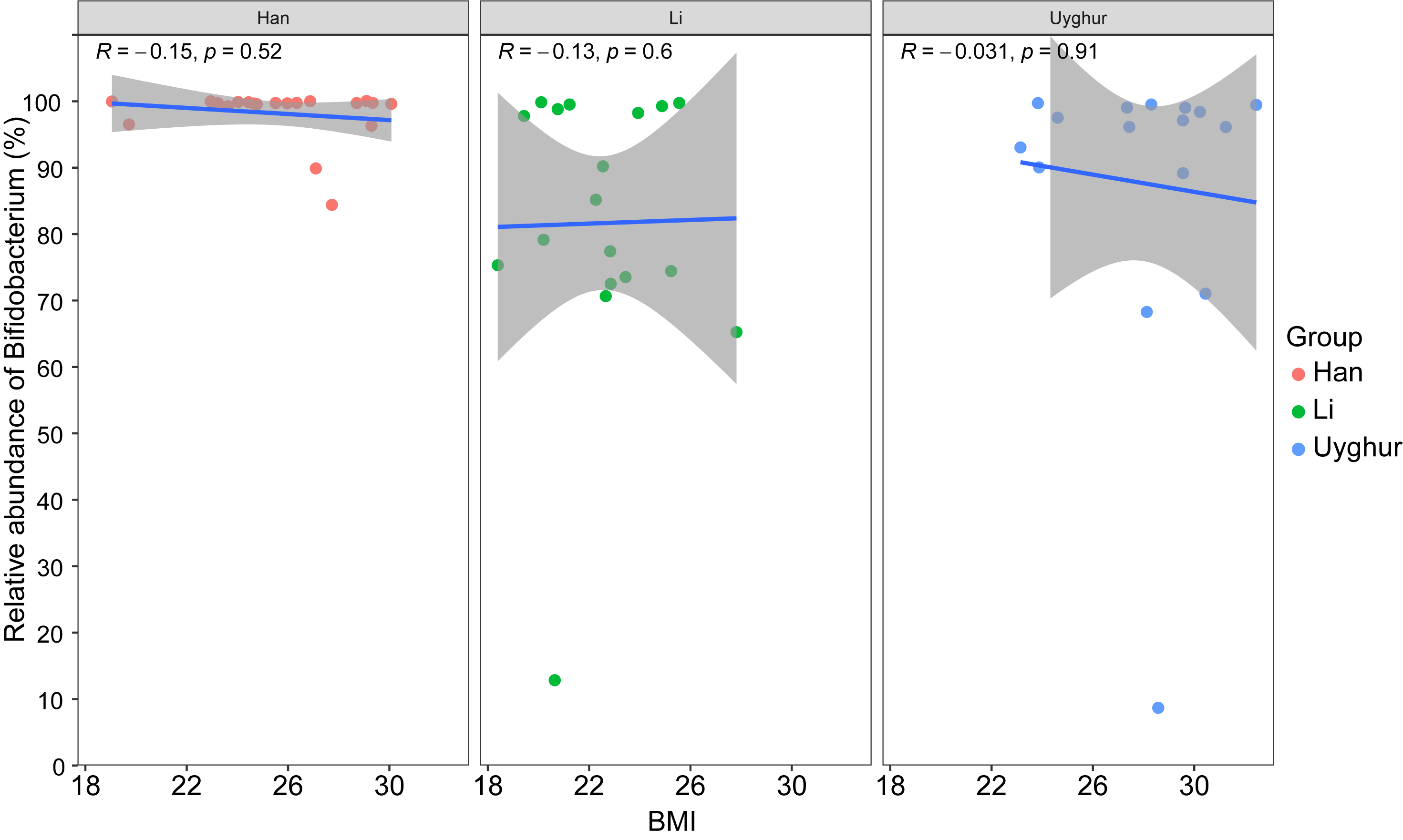


**Table S1.** Primers for MLST analysis.

**Table S2.** Bacterial strains used in this study.

**Table S3.** α diversity of gut microbiota in maternal and infant populations based on 16S rRNA gene.

**Table S4.** Statistical results of significance test of dissimilarity between groups based on 16S rRNA gene.

**Table S5.** α diversity of *Bifidobacterium* in maternal and infant populations profiled by specific-bifidobacterial genes.

**Table S6.** The relative abundance of gut *Bifidobacterium* species identified by specific primers.

**Table S7.** The occurrence of *Bifidobacterium* species and ASV level sharing events based on the *groEL* gene.

**Table S8.** The occurrence of *Bifidobacterium* species and ASV level sharing events based on the *tuf* gene.

**Table S9.** Bacterial composition of mock communities (the relative abundance of each taxon).

**Table S10.** Diet habits of mothers and infants of the three ethnic groups during breastfeeding.
